# Trends in the evolution of intronless genes in *Poaceae*

**DOI:** 10.3389/fpls.2023.1065631

**Published:** 2023-02-16

**Authors:** Yong Chen, Ting Ma, Tingting Zhang, Lei Ma

**Affiliations:** College of Life Science, Shihezi University, Shihezi, Xinjiang, China

**Keywords:** intronless genes, multi-exon genes, retrotransposition, relaxed selection pressure, gene duplication, genome comparison, *Poaceae*

## Abstract

Intronless genes (IGs), which are a feature of prokaryotes, are a fascinating group of genes that are also present in eukaryotes. In the current study, a comparison of *Poaceae* genomes revealed that the origin of IGs may have involved ancient intronic splicing, reverse transcription, and retrotranspositions. Additionally, IGs exhibit the typical features of rapid evolution, including recent duplications, variable copy numbers, low divergence between paralogs, and high non-synonymous to synonymous substitution ratios. By tracing IG families along the phylogenetic tree, we determined that the evolutionary dynamics of IGs differed among *Poaceae* subfamilies. IG families developed rapidly before the divergence of *Pooideae* and *Oryzoideae* and expanded slowly after the divergence. In contrast, they emerged gradually and consistently in the *Chloridoideae* and *Panicoideae* clades during evolution. Furthermore, IGs are expressed at low levels. Under relaxed selection pressure, retrotranspositions, intron loss, and gene duplications and conversions may promote the evolution of IGs. The comprehensive characterization of IGs is critical for in-depth studies on intron functions and evolution as well as for assessing the importance of introns in eukaryotes.

## 1 Introduction

Intronless genes (IGs), which are associated with high transcriptional fidelity, are critical for the regulation of important processes. They not only are a characteristic feature of prokaryotes but also exist in eukaryotes (Jain et al., 2008), representing 2.7%–97.7% of the genes in eukaryotic genomes (Louhichi et al., 2011). Because of the lack of introns, IGs can be more efficiently transcribed than multiexon genes (MEGs) with at least one intron (Souza, 2003; Chorev and Carmel, 2012; Savisaar and Hurst, 2016). Moreover, they encode proteins belonging to various large families, including G protein–coupled receptors, olfactory receptors, histones, transcription factors, and the regulators of signal transduction and development (Gentles and Karlin, 1999; Zhang and Firestein, 2002; Sakharkar et al., 2005). IGs are a valuable genetic resource for in-depth studies of intron function and evolution. For example, they may be used to clarify the importance of introns in eukaryotes.

Many studies have explored the origin of IGs in different evolutionary periods. Some studies have attempted to make general conclusions regarding IGs in all eukaryotes by comparing deeply divergent species (Csűrös et al., 2007; Grau-Bové et al., 2017). However, the long-branch attraction effect occurs when widely divergent taxa or clades have many state changes, which can lead to incorrect trees and inappropriate statistical analyses (Felsenstein, 1978). In contrast, some studies focused on specific species and provided detailed insights into the target organism (Jain et al., 2008; Yan et al., 2014), but their findings may not be applicable to other species because some evolutionary events may be restricted to certain periods, whereas others may be specific to particular taxa (Cohen et al., 2012). For example, many cases of intron gain and loss have been investigated, but it remains unknown when these changes occurred during evolution (Knowles and McLysaght, 2006; Carmel et al., 2007; Roy and Penny, 2007). Meanwhile, different evolutionary events may have occurred simultaneously within the same organism. For example, some genomes may contain both intronless and intron-rich copies of a particular gene (Liu et al., 2021).

*Poaceae* evolved into a distinct taxon 50–70 million years ago (Mya). Its closely related and well-studied subfamilies (Stanley, 1999) have enabled researchers to confidently determine the phylogenetic relationships within clades (Soreng et al., 2015; Soreng et al., 2017; Soreng et al., 2022). *Poaceae* is also one of the most ecologically and economically important plant families, accounting for 25%–45% of the vegetation worldwide (Thomasson, 1980). Studying IGs in *Poaceae* will clarify the evolutionary path that resulted in grasses becoming one of the major plant families on Earth. In the present study, we focused on the clades of *Poaceae* and tracked the parallel large changes in IGs in the genomes of the related species *Brachypodium distachyon* (Fox et al., 2013), *Eragrostis curvula* (Carballo et al., 2019), *Leersia perrieri* (Loera-Sánchez et al., 2022), *Oryza sativa* (Kawahara et al., 2013), *Panicum hallii* (Lovell et al., 2018), *Setaria italica* (Bennetzen et al., 2012), *Sorghum bicolor* (Paterson et al., 2009), *Zea mays* (Hufford et al., 2021), and *Oropetium thomaeum* (VanBuren et al., 2015). We explored the evolutionary branches with minimal changes in clades or specific organisms. Furthermore, we precisely paired paralogous gene copies in each genome according to the number of introns to assess the effect of intron density on genes, thereby clarifying the evolutionary and genetic mechanism associated with IGs. Finally, we propose a model of the origin and expansion of IG families. The findings of our study have clarified the evolutionary trajectories of IGs in *Poaceae*.

## 2 Results

### 2.1 Paralogy of intronless genes

We selected nine thoroughly sequenced genomes to represent *Poaceae*, of which three were from species in the *Pooideae* and *Oryzoideae* (PO) clade and six were from species in the *Chloridoideae* and *Panicoideae* (CP) clade. The proportion of IGs varies among grasses, ranging from 13% to 30% (**Supplementary Figure S1**). Furthermore, IGs differ in terms of copy numbers. For example, there are many distinct clusters of IGs in the gene family tree of the Rx_N-terminal domain of *E. curvula* and Cytochrome_P450 of *S. bicolor* (**Supplementary Figure S2**).

**Figure 1:**
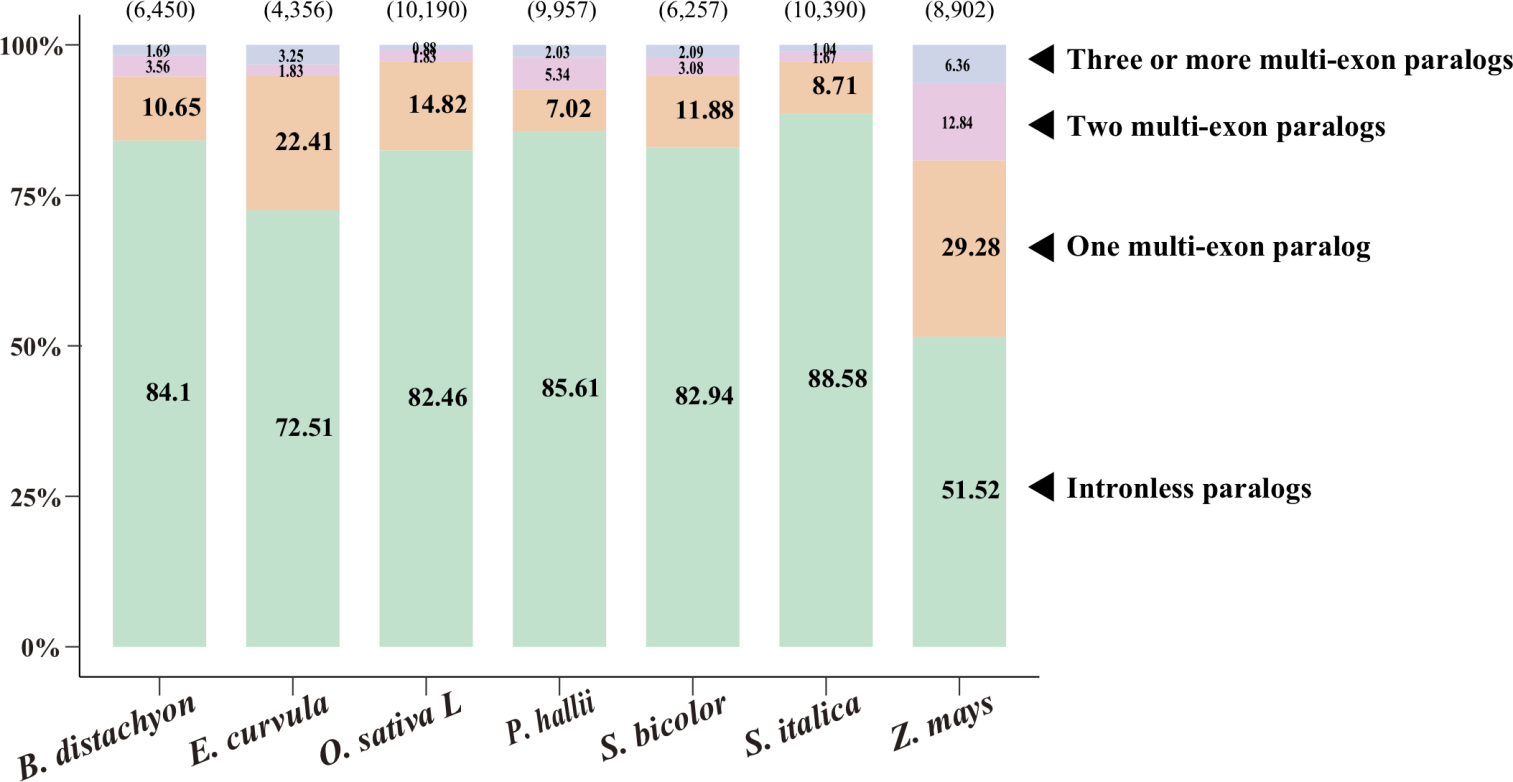
Paralogy of intronless genes (IGs). Coding sequences of IGs were aligned to their paralogs, including intronless paralogs and one, two, and three or more multiexon paralogs. The number of IGs is indicated in parentheses above each bar, whereas the percentages of mapped IGs are provided on the bar. The source data are provided in **Supplementary Table S1**.

To investigate paralogy, we aligned the coding sequences of IGs to their corresponding paralogs (**Figure 1**, **Supplementary Table S1**; see Methods). Of the 4,356–10,390 IGs in the nine species, most (51%–89%) were mapped to their intronless paralogs, but 7%–22% were mapped to one multiexon paralog and a few were mapped to two or more multiexon paralogs. Accordingly, intronless paralogs appear to have diverged substantially from multiexon paralogs, making it difficult to trace them back to multiexon paralogs. This phenomenon reflects the relatively high substitution rates for these genes (Carvunis et al., 2012). This result is also consistent with the gene family trees (**Supplementary Figure S2**), in which some intronless paralogs were clustered together and some were even nested under branches with multiexon paralogs.

### 2.2 Retrotransposon density around intronless genes

**Figure 2:**
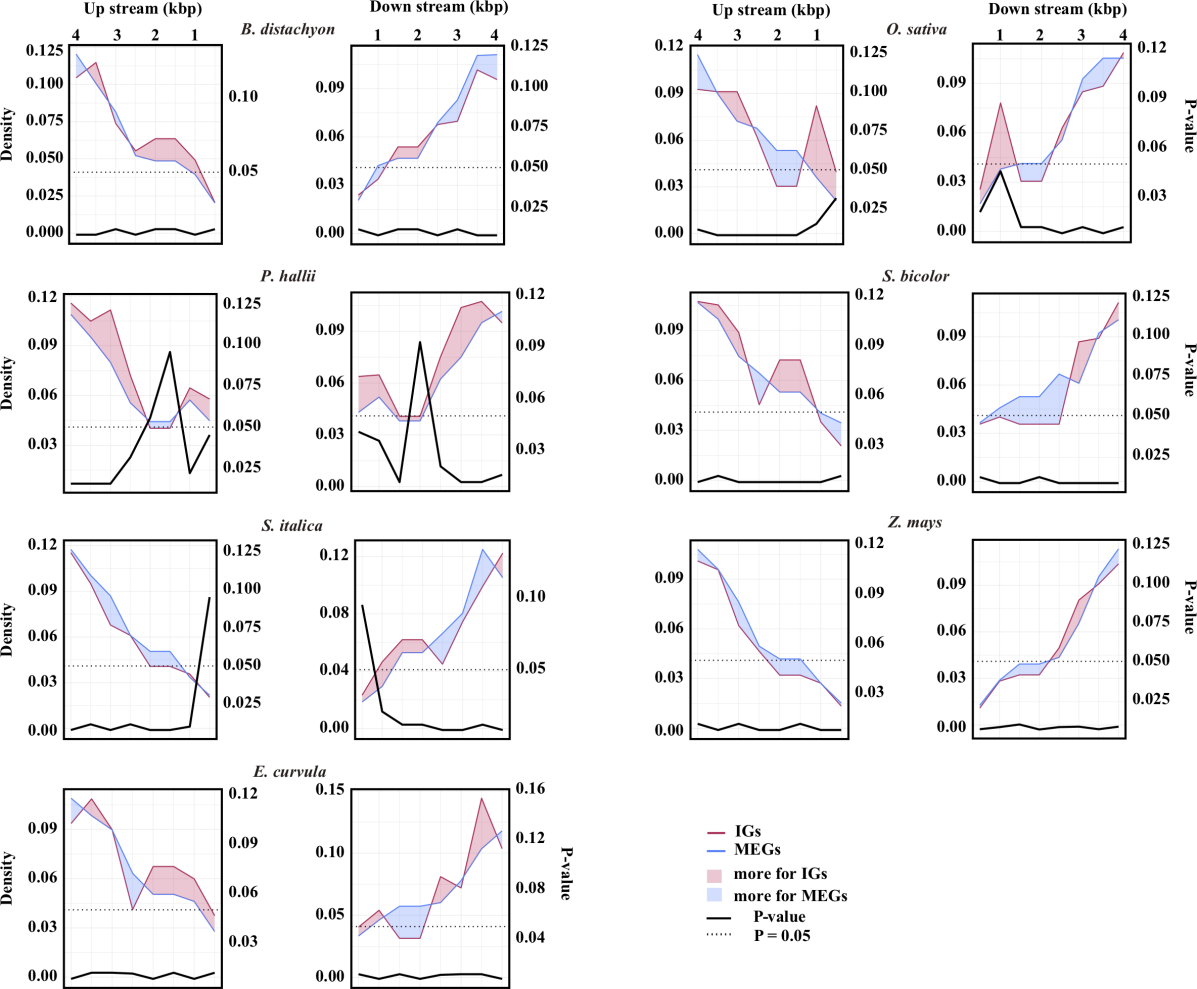
Retrotransposon landscape surrounding genes. Retrotransposon density around IGs (red line) or multiexon genes (MEGs, blue line). The number of retrotransposons was determined in 100-bp windows and averaged for every 500-bp window from the transcription start site to 4 kb upstream and from the transcription termination site to 4 kb downstream. The data were normalized according to the number of IGs or MEGs. The left y-axis shows the normalized density. The red area indicates that there are more retrotransposons for IGs than for MEGs in that region, whereas the blue area indicates the opposite. The source data are provided in **Supplementary Table S2**. The differences in the number of retrotransposons between the IGs and the MEGs were assessed by a two-sided paired Wilcoxon sign test. The right y-axis shows the *P*-value.

The retrotransposable element composition of the flanking sequences differed between IGs and MEGs (**Figure 2**; **Supplementary Table S2**). In *E. curvula*, *O. sativa*, and *P. hallii*, more retrotransposons were detected in the regions immediately surrounding IGs than in the corresponding regions of MEGs. In *B. distachyon*, the region immediately upstream of IGs had more retrotransposons than the corresponding region of MEGs. In contrast, the region immediately downstream of IGs had more retrotransposons than the corresponding region of MEGs in *S. italica*. In *Z. mays*, the 500 bp region immediately surrounding IGs had a few more retrotransposons than the corresponding region of MEGs.

### 2.3 Evolutionary trajectories of intronless gene families

**Figure 3:**
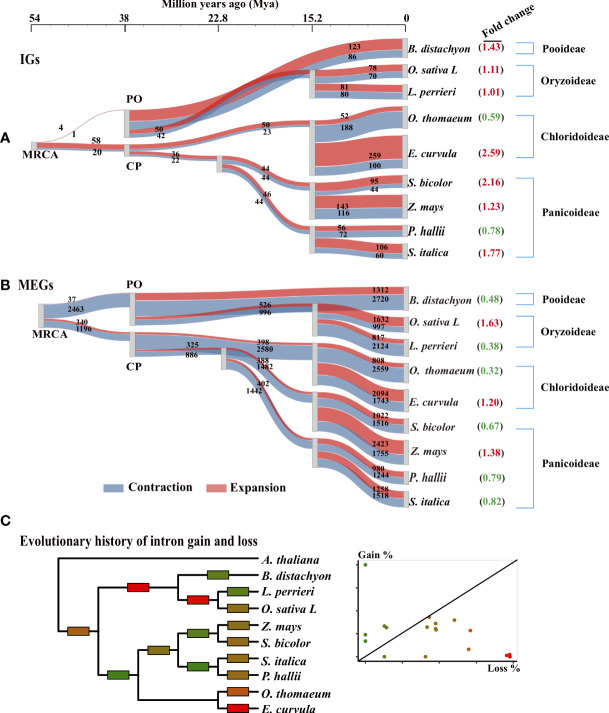
Evolutionary trajectory of IGs in *Poaceae*. **(A, B)** Gene family expansion and contraction along the phylogenetic tree for IG and MEG families. The 242 single-copy orthologous gene groups of nine *Poaceae* species were used to reconstruct the phylogenetic tree with two outgroups (*Cinnamomum chinensis* and *Arabidopsis thaliana*; not shown). The most recent common ancestors (MRCAs) of *Poaceae* represent the tree root. Three species are from the *Pooideae* and *Oryzoideae* (PO) clade and six are from the *Chloridoideae* and *Panicoideae* (CP) clade. A Sankey diagram shows the flow rate of gene families along the tree, where the width of each element is proportional to the expansion (red) and contraction (blue) of the gene family. The number of genes in each family is displayed on the element. The fold change (expansion to contraction) in the number of genes in each family is shown in parentheses. **(C)** Evolutionary history of intron gain and loss was reconstructed according to the Markov method (Csűrös et al., 2011). Edges are colored according to the relative amount of intron gain and loss as indicated in the scatter plot in which each point corresponds to an edge in the tree. Gain% is the percentage of introns gained in the given lineage from the parent node. Loss% is the percentage of the parental introns that were lost within the same lineage.

To study the evolutionary trajectories of IGs in *Poaceae*, we reconstructed a tree representing the gene family history of grass lineages (**Figure 3**). The 242 single-copy orthologous gene groups of the nine *Poaceae* species were selected to reconstruct this phylogenetic tree with two outgroups (*Cinnamomum chinensis* and *Arabidopsis thaliana*). A family of IGs in a genome was defined as a paralogous group in which 50%–70% of the genes did not contain introns; the upper limit was set to exclude overly homogeneous genetic structures that would have adversely affected reliability. Orthologous groups of IGs should be present in 60% of the species. The 548 IG families identified in modern species were subsequently traced individually to the most recent common ancestor (MRCA).

To visualize the origin of IGs, we used a Sankey diagram to present the flow rates of gene families along the tree (**Figure 3A**). Over time, the IG families gradually emerged. For example, the MRCA had only approximately 80 IG families, but some modern species had more than 500 IG families. Furthermore, the increase in the number of IGs differed slightly between the PO and CP clades (**Figure 3A**). The number of IGs increased quickly before PO divergence and then subsequently increased slowly. For example, in the clade with *O. sativa* and *L. perrieri*, the IG families of the progeny of the PO ancestor were approximately 18 times larger (50 + 42 vs. 4 + 1) than that of the MRCA approximately 38 Mya. Moreover, the IG families of the modern *O. sativa* and *L. perrieri* species were approximately 1.75 times larger (78 + 70 vs. 50 + 42) and 1.60 times larger (81 + 80 vs. 50 + 42) than that of their *Oryzoideae* ancestor (one progeny of the PO ancestor), respectively. The IG families in the CP clade expanded slowly over time. For example, the earlier (approximately 38 Mya) and most recent (approximately 22 Mya) ancestors of *Panicoideae* in the CP node had a similar number of IG families.

### 2.4 Bias of expansion over contraction

The trajectories in the tree revealed the expansion and contraction history of the IG families (**Figure 3A**). The number of expanded IG families at the MRCA was greater than the number of contracted IG families at the node (PO: four expanded and one contracted; CP: 58 expanded and 20 contracted). However, the reverse trend was observed for the MEGs (PO: 37 expanded and 2,463 contracted; CP: 34 expanded and 1,196 contracted). For most lineages, there were more expanded IG families than contracted IG families (**Figure 3A**). Conversely, there were fewer expanded MEG families than contracted MEG families (**Figure 3B**), especially during species divergence approximately 15 Mya. The IG families in seven of the nine (78%) modern genomes tended to expand rather than contract, but the MEG families in six of the nine (67%) modern genomes contracted substantially.

### 2.5 Duplication of intronless genes

**Figure 4:**
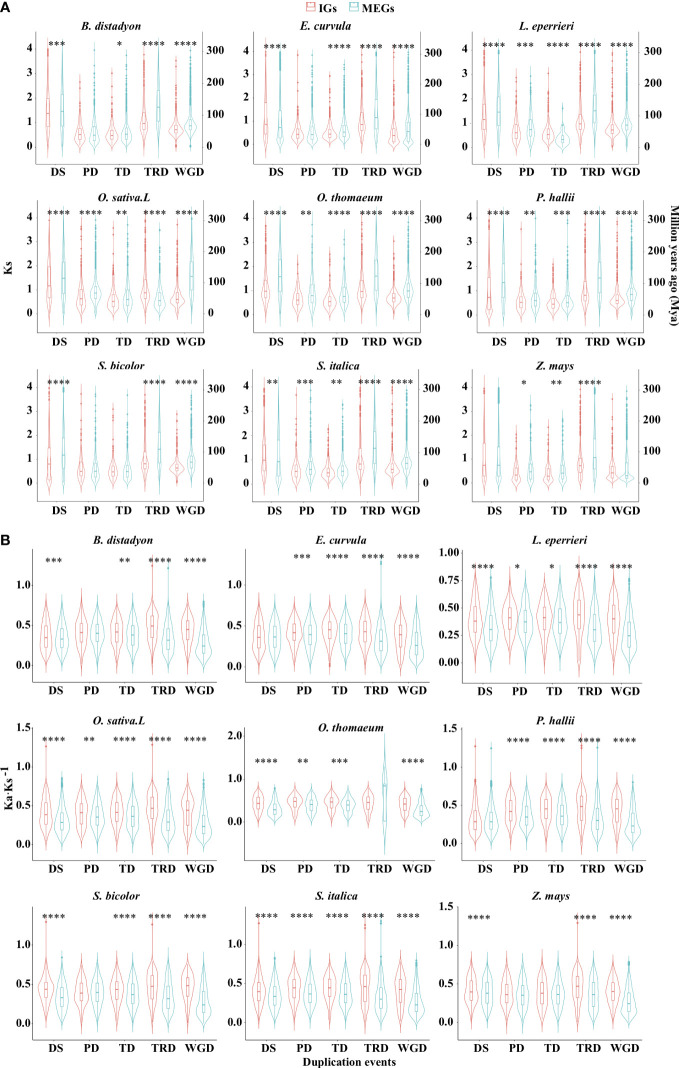
Distribution of Ks **(A)** and Ka·Ks^−1^ **(B)** values for the IGs and MEGs derived from gene duplications. Dispersed (DSD), proximal (PD), tandem (TD), transposed (TRD), and whole-genome (WGD) duplications. Violin plots present the kernel probability density distribution. The overlaid boxplots present the data range and the distribution spread. The horizontal inner line represents the median value. The bars range from the 25th (bottom) to the 75th (top) percentile and the vertical lines represent 95% confidence intervals. Divergence time **(T)** was calculated as Ks·(2r)**^−1^**, where r is the neutral substitution rate (6.50 × 10^−9^). Asterisks represent significant differences (two-sided paired Wilcoxon sign test: *P < 0.05, **P < 0.01, ***P < 0.001, ****P < 0.0001). The source data are provided in **Supplementary Table S3**.

To elucidate the expansion patterns, genes were assigned to the following five gene duplication events: tandem duplication (TD), proximal duplication (PD), dispersed duplication (DSD), transposed duplication (TRD), and whole-genome duplication (WGD) (**Supplementary Table S3**; **Figure S3**). For most events in the nine examined species, the median synonymous substitution rate (Ks) was significantly lower for the IGs than for the MEGs (**Figure 4A**; **Supplementary Table S3**; *P* < 0.05, two-sided paired Wilcoxon sign test). Specifically, of the five duplication events, four in *O. sativa*, four in *B. distachyon*, three in *E. curvula*, four in *L. perrieri*, five in *O. thomaeum*, five in *P. hallii*, three in *S. bicolor*, four in *S. italica*, and three in *Z. mays* had such a trend. Furthermore, the analysis of the nine species revealed that DSD for five species, PD for six species, TD for seven species, TRD for eight species, and WGD for eight species also had such a trend. According to the molecular evolutionary clock and the neutral theory of molecular evolution, some duplication events may have occurred later for IGs than for MEGs in some species. Because duplicated genes result in new genetic material during the evolution of plants (Brosius, 1991), these results indicate that gene-duplication events were crucial for the evolution of IGs.

**Figure 5:**
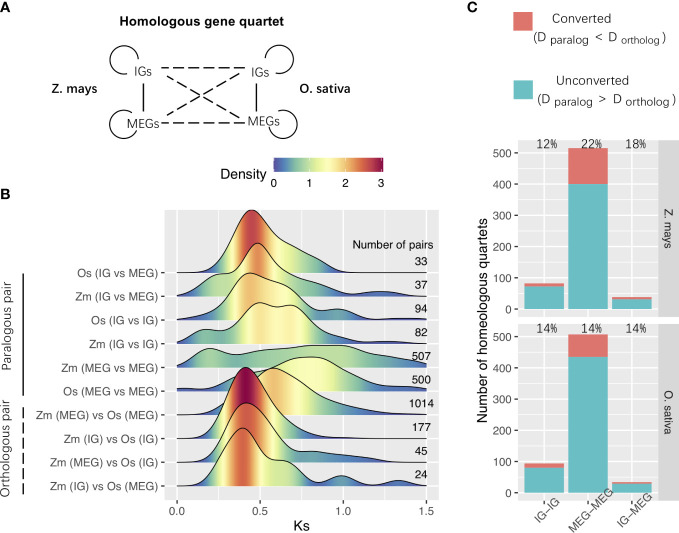
WGD-derived homologous gene quartets. **(A)** Relationships among the homologous gene quartets for *Zea mays* and *O. sativa*. Solid and dashed lines represent the paralogous and orthologous relationships between the WGD-derived genes, respectively. **(B)** Ks distributions for the WGD-derived gene pairs among homologous gene quartets. The number of pairs is shown to the right of the distribution panel. **(C)** Gene conversion. Conversion rates are provided in the panel. Zm, *Z. mays*; Os, *O. sativa*; IG, Intronless gene; MEG, multiexon gene; D, distance.

We then analyzed WGD-derived homologous genes in *Z. mays* and *O. sativa*. Using previously described methods (Sawyer, 1989; Wang et al., 2007; Qiao et al., 2019), we identified 635 WGD-derived homologous gene quartets comprising two paralogs in the species of interest and their respective orthologs in outgroup species (**Figure 5A**). The densest Ks peaks were at higher Ks values for paralogous pairs than for orthologous pairs (**Figure 5B**). This indicates that most paralogs in each species were more similar to their respective orthologs in the other species than to each other, implying that most of them were derived from duplication events that occurred before species divergence.

Interestingly, WGD events affected the whole genome (Adams and Wendel, 2005), but the Ks density peaks for WGD differed between IGs and MEGs (**Figure 4A**). This difference may be at least partly related to WGD-related “erosion.” Earlier research revealed that WGD events are commonly followed by the loss of most duplicated genes over a few million years (Lynch and Conery, 2000; Weisman et al., 2020). This loss occurs in an episodic manner (Bowers et al., 2003; Jiao et al., 2014). Successive WGD events are often separated by tens of millions of years, preventing them from providing a continuous supply of variants available for adaptations to changing environmental conditions. The erosion may be associated with genomic modifications (e.g., chromosomal rearrangement, gene loss, gene conversion, subgenome dominance, and a divergent expression of duplicated copies) (Adams and Wendel, 2005; Jiao and Paterson, 2014; Wendel et al., 2016). The erosion was revealed by the higher Ka·Ks^−1^ values for IGs than for MEGs in most species (**Figure 4B**).

We also investigated the gene conversion–related erosion effect on WGD-derived duplicates. Gene conversions can increase the number of low-divergence paralogs and affect the evolution of various multigene families (Sawyer, 1989; White and Crother, 2000; Mondragon-Palomino and Gaut, 2005). Gene conversions reportedly occurred after the species divergence when the paralogs were more similar to one another than to their cross-species orthologs (Wang et al., 2007). We estimated the gene conversion rates of duplicated genes derived from *Poaceae* ρ-WGD events in *Z. mays* and *O. sativa.* The Ks values were used to represent the evolutionary distance, and a bootstrap test was performed to evaluate the significance of putative gene conversions. We determined that paralogous gene were affected by gene conversions (**Figure 5C**). For example, the conversion between MEGs and MEGs occurred most frequently in *Z. mays* (**Figure 5C**). These results imply that gene conversion may be one of the mechanisms that alter paralog compositions and Ks distributions after WGD events.

### 2.6 Relaxed selection pressure on intronless genes

Although most paralogous gene pairs for the five duplication events had Ka·Ks^−1^ values less than 1, the selection pressure was more relaxed for the IGs than for the MEGs for most of the duplication events in the nine species (*P* < 0.05, two-sided paired Wilcoxon sign test; **Figure 4B**). For example, of the five duplication events, five in *O. sativa*, four in *B. distachyo*n, four in *E. curvula*, five in *L. perrieri*, four in *O. thomaeum*, four in *P. hallii*, four in *S. bicolor*, five in *S. italica*, and three in *Z. mays* tended to be associated with relaxed selection pressure. In addition, of the nine species, DSD for seven species, PD for six species, TD for eight species, TRD for eight species, and WGD for nine species exhibited the same tendency. These findings suggest that IGs derived from most duplication events were under relaxed selection pressure.

### 2.7 Intron loss in *Poaceae*

**Figure 6:**
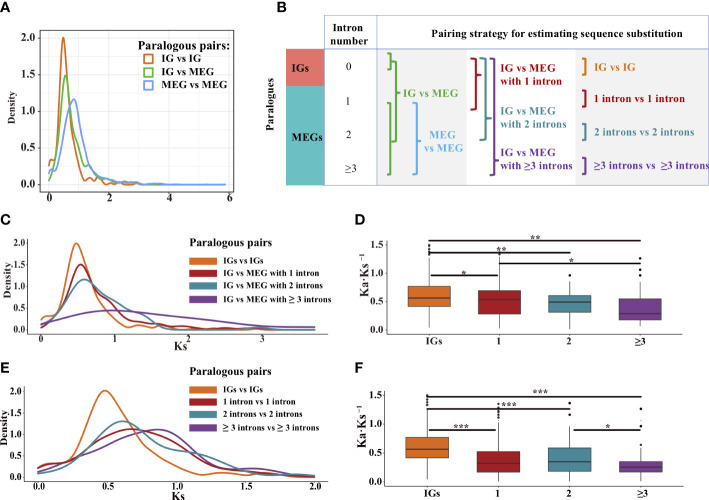
Distribution of the Ks and Ka·Ks^−1^ values for the paralogous pairs in rice. **(A)** Density plot of the Ks values for three paralogous sets: intronless [IG vs. IG], transition (IG vs. MEG), and multiexon (MEG vs. MEG). **(B)** Paralogous pairing strategy. **(C, D)** Paralogous pairs between IGs and MEGs with one, two, and three or more intron(s). **(E, F)** Paralogous pairs of genes with the same number of introns. The boxplots present the median, upper, and lower quartiles and 95% confidence intervals for Ka·Ks^−1^. Points indicate outliers in the data. Asterisks represent significant differences (two-sided paired Wilcoxon sign test: *P < 0.05, **P < 0.01, and ***P < 0.001).

Using the Markov model (Csűrös et al., 2011), we reconstructed the evolutionary history of intron gain and loss. Our analysis indicated that intron loss was the main process in most *Poaceae* lineages (**Figure 3C**). To further explore how gene structures evolved, we grouped paralogous gene pairs into the following three sets: intronless (IG vs. IG), transition (IG vs. MEG), and multiexon (MEG vs. MEG) (**Figure 6B**). The first and third sets were used to detect the divergence among intronless paralogs or multiexon paralogs. The second set (IG vs. MEG) reflected the transition state between intronless and MEGs. The Ks distribution trends varied among the three sets (**Figure 6A**, **Supplementary Figure S4**). For most species, the Ks values were typically lower for the intronless (IG vs. IG) set, whereas the Ks values tended to be higher for the multiexon (MEG vs. MEG) set, implying that most intronless paralogs were created later than the multiexon paralogs. The transition state links the conversion of IGs into MEGs. Hence, it is possible that MEGs may evolve into IGs following the loss of introns.

To further clarify how introns are lost, gene families at the transition state were divided into four subfamilies of genes containing zero, one, two, and three or more intron(s) (**Figure 6B**). For rice, the Ks values for the MEGs tended to decrease as the number of introns decreased (**Figure 6C**), implying that the introns were gradually lost over time. In addition, the purifying selection pressure was weaker for the IGs than for the multiexon groups (*P* < 0.05, the two-sided paired Wilcoxon sign test on Ka·Ks^−1^ values; **Figure 6D**). These phenomena were also observed for some other species (**Supplementary Figure S5**). Thus, compared with younger genes, older genes may contain more introns. This is because the exon–intron architecture increases genetic complexity and highly complex genes develop over a long evolutionary period (Carvunis et al., 2012; Werner et al., 2018). Therefore, we speculated that through the loss of introns, intron-rich genes evolved into intron-poor genes, which then became IGs.

To further elucidate the origin of IGs, we evaluated the divergence among paralogous gene pairs containing the same number of introns (**Figure 6E**). In rice, the Ks values were lower for the genes with one or two introns than for the genes with three or more introns (**Figure 6E**). In addition, the median Ka·Ks^−1^ values were higher for the IGs than for the MEGs (**Figure 6F**, **Supplementary Figure S6**), suggestive of a gradual relaxation of the selection pressure during the transition from an intron-rich state to an intron-poor state. The decrease in the number of introns may have had beneficial effects on some gene functions, thereby enhancing the ability of organisms to cope with biotic and abiotic stresses (Chen et al., 2021).

### 2.8 Gene ontology enrichment analysis

**Figure 7:**
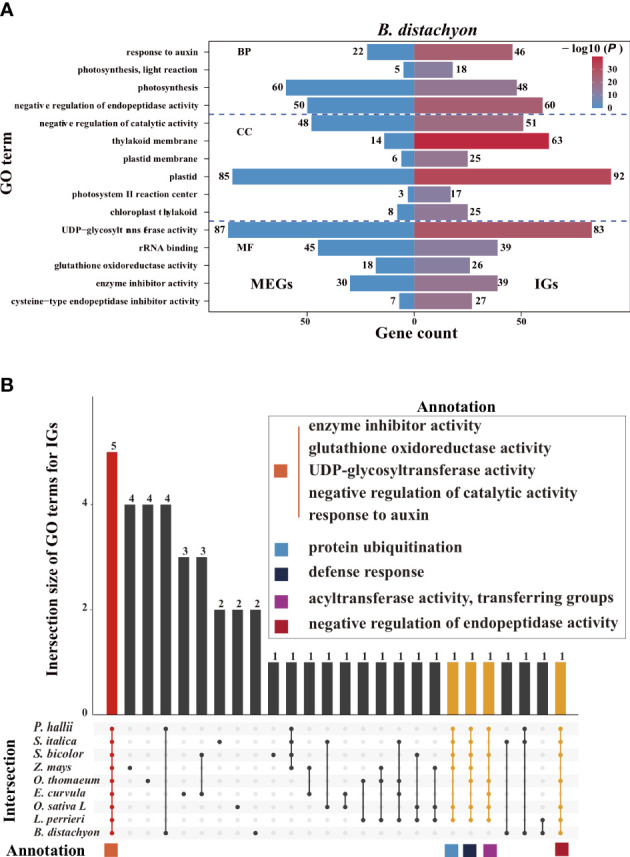
Enriched Gene Ontology (GO) terms. **(A)** Top 15 enriched GO terms among the *B. distachyon* (IGs, right) and MEGs (left). The color scale represents the *P-* values for the enrichment test. The number next to each bar represents the gene count for that GO term. **(B)** Intersection of the top 15 enriched GO terms for the IGs in nine *Poaceae* species. Bar colors represent the three subsets where all 9 (red), 6–8 (yellow), and 1–5 (black) species intersect. The common GO terms in at least six species are shown in the panel with colored squares.

We performed a Gene Ontology (GO) enrichment analysis to assess whether IGs and multiexon genes have different biological functions (**Figure 7A**, **Supplementary Figure S7**). Significantly enriched GO terms were identified for the IGs but not for the MEGs, reflecting the functional diversity between the two gene types in *Poaceae*. Some of the enriched GO terms assigned to the IGs are commonly enriched among the genes in *Poaceae* genomes (**Figure 7B**), including three molecular function terms (enzyme inhibitor activity, glutathione oxidoreductase activity, and uridine diphosphate (UDP)-glycosyltransferase activity) and two biological process terms (negative regulation of catalytic activity and response to auxin). These genes are involved in fundamental enzymatic processes that affect plant growth, biotic and abiotic stress responses, and the regulation of gene expression. Additionally, several other GO terms, including protein ubiquitination, defense response, acyltransferase activity, and transfer group, were also common among the genes in most genomes.

### 2.9 Low intronless gene expression levels

**Figure 8:**
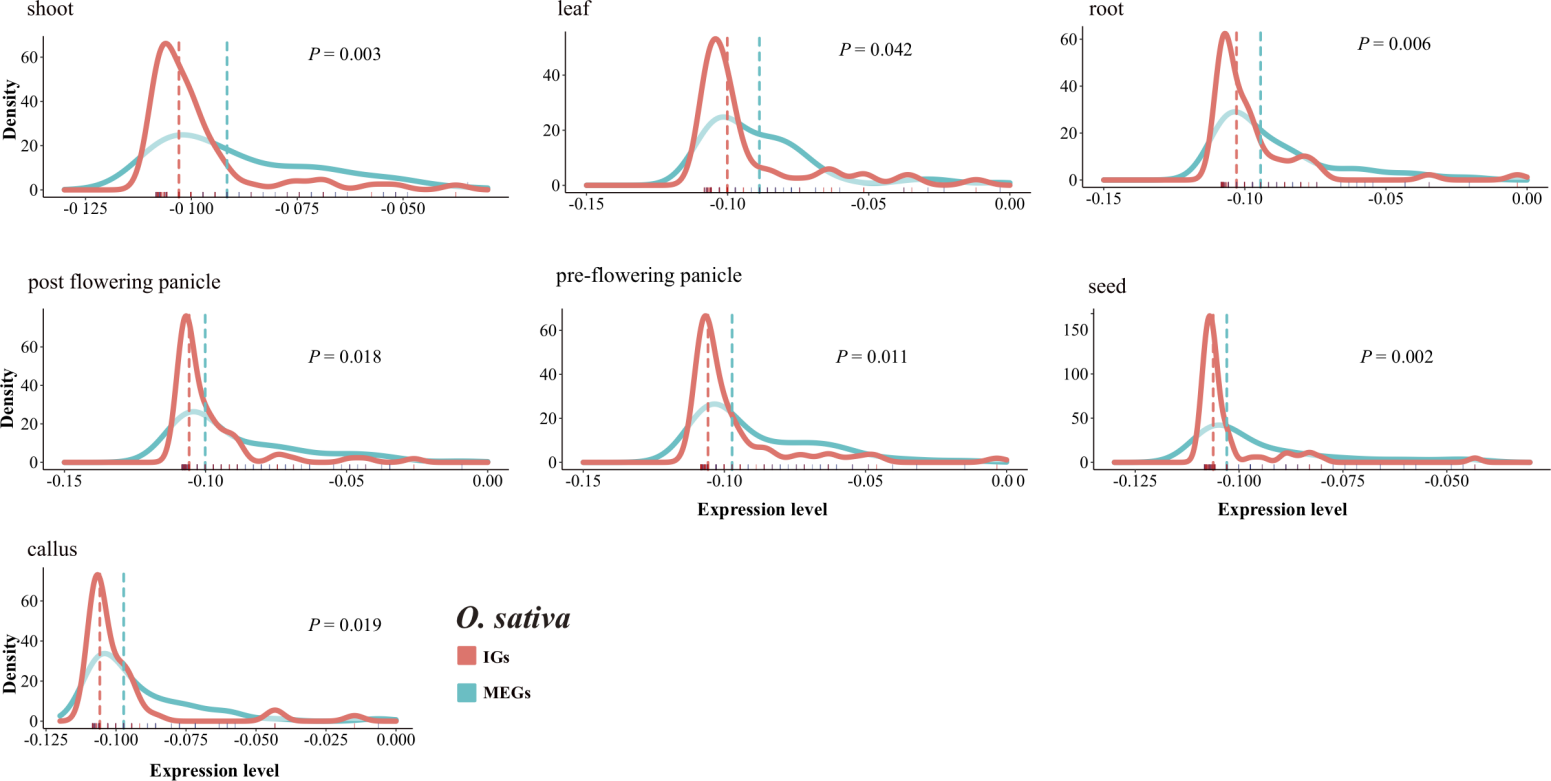
Comparison of the IG and MEG expression levels in rice tissues. The dotted line represents the median normalized expression level. The rug on the horizontal axis shows the density. The *P*-value for a two-sided paired Wilcoxon sign test is provided.

We compared the transcriptional patterns of IGs and MEGs using expression data for rice and maize. A total of 189 rice genes (94 MEGs and 95 IGs) and 125 maize genes (80 MEGs and 45 IGs) annotated with the GO term UDP-glycosyltransferase activity were investigated. Overall, the IGs were expressed at lower levels than the MEGs (**Figure 8**, **Supplementary Figure S8**). More specifically, in seven rice tissues, the median expression level was much lower for the IGs than for the MEGs (*P* < 0.05, two-sided paired Wilcoxon sign test) (**Figure 8**). The same trend was observed for 6 of 10 maize tissues (**Supplementary Figure S8**). Furthermore, in all seven rice tissues, the IG expression levels had higher peaks and narrower expression widths than the MEG expression levels (**Figure 8**), which is similar to the findings of an earlier study (Sakharkar et al., 2005). These results may reflect the limited diversity in the expression levels of intronless isoforms. This is also in accordance with the results of another previous study (Werner et al., 2018) that concluded that newer genes are expressed at lower levels than older genes. The observed differences in expression levels are consistent with the potential neofunctionalization (or pseudogenization) of newer genes that are evolving neutrally.

## 3 Discussion

Plants have a wide variety of exon–intron frameworks that differ in terms of the number, length, and structure of introns. Some studies examined the diversity in all eukaryotes, including plants, to explore the sources of these differences (Csűrös et al., 2007; Grau-Bové et al., 2017), which resulted in broad conclusions for highly diverse species. However, extremely large evolutionary distances may result in long-branch attraction effects. Moreover, the diversity among a limited number of species may be insufficient for statistical analyses. In the present study, we focused on the *Poaceae* clade to generate important insights into this plant family. The study findings may be useful for tracking parallel large changes occurring in related organisms.

Several earlier studies suggested that intron loss has been the dominant evolutionary trend rather than intron gain (Stajich and Dietrich, 2006; Carmel et al., 2007; Worden et al., 2009), but it remains unclear why gene families in a given genome simultaneously contain both intronless and intron-rich gene copies. In the current study, we tracked the evolutionary history of gene families in *Poaceae* lineages, which provided relevant insights into the origin, expansion, and contraction of IG and MEG families over a particular timescale. In addition, we carefully paired paralogous copies in each genome to assess their diversity and examine the timing of the associated gene duplication events.

### 3.1 Intronless genes may be derived from multiexon genes that have lost introns

After surveying the genomes of representative species, we revealed that the median Ks values were lower for IGs than for MEGs (**Figures 4**, **6**) in some species. Similar results were obtained in previous studies (Zhong et al., 2018; Zhu et al., 2018; Liu et al., 2021). This Ks pattern suggests that IGs may be younger than MEGs. The same trend was also observed for the gene families containing both intronless and multiexon paralogs. In some species, a decrease in the number of introns in genes was associated with a decrease in the peak Ks value (**Figures 6A, C**). In other words, compared with the older genes, younger genes have fewer introns. Furthermore, a decrease in the number of introns decreased the synonymous variations (**Figure 6E**), indicative of a general trend toward intron loss. Moreover, in genomes, the proportion of IGs was lower than that of MEGs (**Supplementary Figure S1**), which is consistent with previously reported findings (Yan et al., 2014). Thus, compared with MEGs, IGs have a shorter history and are less abundant in genomes. It is possible that IGs originated from MEGs that lost introns.

### 3.2 Splicing, reverse transcription, and retrotransposition

Because of alternative splicing, the number of introns for a given gene varies from isoform to isoform (Cazorla et al., 2000). In addition, genes from a large family are usually scattered throughout the genome and may vary regarding the number of introns (Liu et al., 2014). These two phenomena may be related. The spliceosome processes the primary messenger ribonucleic acid (mRNA) sequence of genes, and it is influenced by gene promoters, cellular molecules, and other signals (Spellman et al., 2007), which ultimately affects which exons are included in the final mRNA. Alternative splicing can lead to changes in protein size (e.g., the inclusion/exclusion of specific regions). These alternatively spliced sequences may be reverse-transcribed to DNA by reverse transcriptases (Esnault et al., 2000). The DNA fragments are then inserted into the original chromosome at a different location or into a different chromosome *via* recombination with the assistance of repetitive sequences or transposable elements. Our results clearly show that the abundance of retrotransposable elements near the gene body is greater for IGs than for MEGs in some species (**Figure 2**), implying that the transposition to a new chromosomal position occurs more easily for IGs than for MEGs. Spliced mRNAs lack introns, which means that recombinations involving reverse transcribed copies will result in intron-poor genes. Additionally, IGs are mainly derived from the transposition of duplicates (**Figure 4**), which is mediated by DNA- or RNA-based transposable elements and leads to gene pairs consisting of ancestral and novel loci (Wolfe, 2007; Wang. et al., 2012).

### 3.3 Retrotransposition and duplication may drive the increase in the number of intronless genes

In the current study, IGs were included in many distinct clusters in the gene family tree (**Supplementary Figure S2**). Related IGs may arise from the duplication of a “seed” gene. We speculated that retrotransposition and duplication events may be among the main drivers of the substantial increase in the number of IGs in *Poaceae*. Duplication events result in a dramatic increase in the number of genes (Clark and Donoghue, 2018) and are closely related to environmental changes, such as geological expansion (Barker et al., 2016) and temperature fluctuations (Smith et al., 2017). Hence, they were crucial for the stress resistance of *Poaceae* and facilitated species expansion.

### 3.4 Relaxed selection pressure is conducive to intronless gene evolution

The selection pressure following gene duplication events dictates whether genes are retained or lost (Cheng et al., 2018). Gene copies produced by duplication accumulate sequence changes, and, in many cases, the accumulation is highly heterogeneous. Copies then gradually deviate from their paralogs (Holland et al., 2017). Compared with MEGs, the larger Ka·Ks^−1^ values of IGs indicate that they were exposed to relaxed selection pressure (**Figures 4**, **6**). The loss of introns over time has resulted in more efficient transcription (Chen et al., 2021). Earlier research confirmed that transcriptional efficiency increases as the transcript length decreases (Stanley, 1999).

### 3.5 Model of the origin and evolution of intronless and intron-poor genes

**Figure 9:**
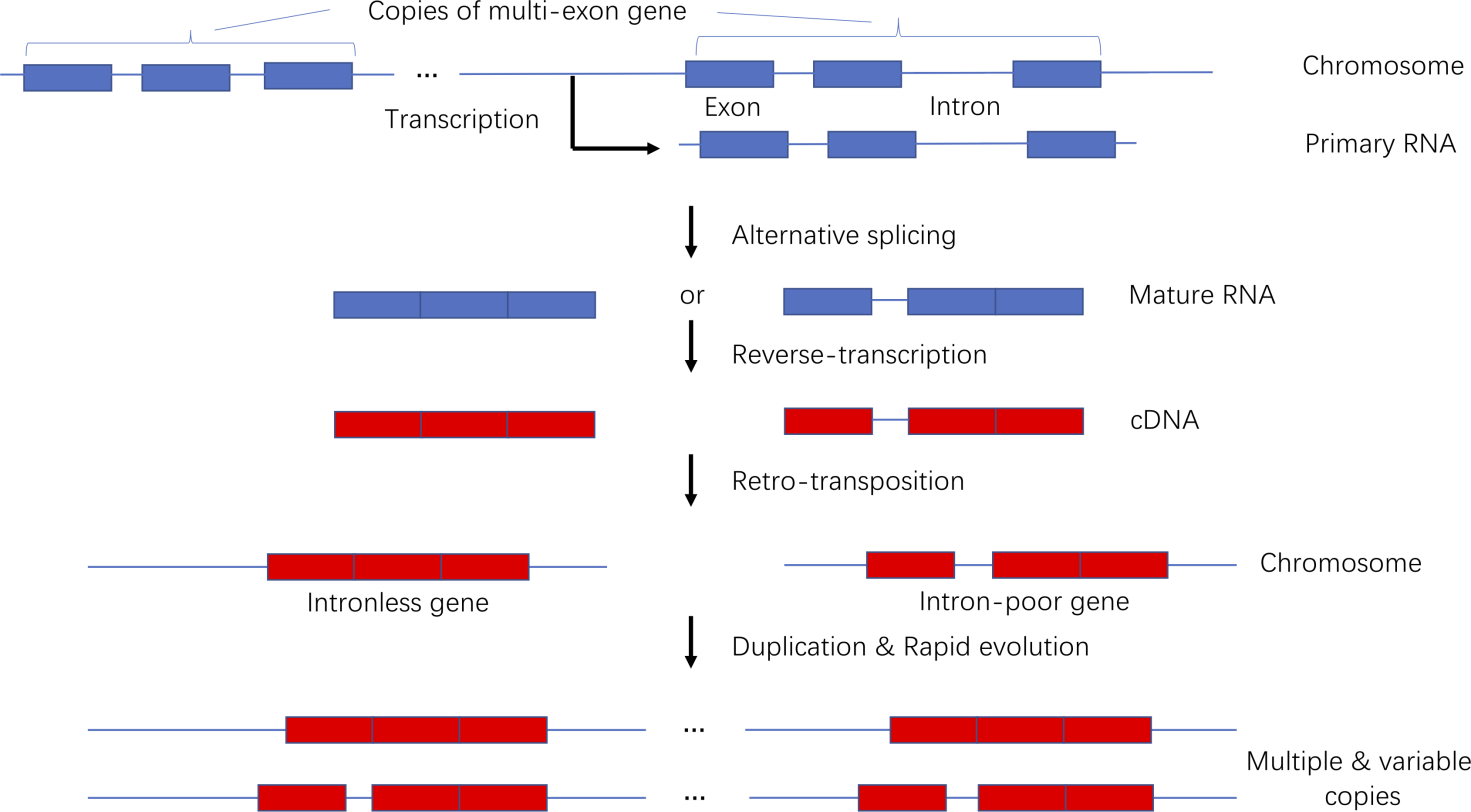
Model of the origin and evolution of intronless and intron-poor genes.

We developed a model presenting the origin and evolution of intronless and intron-poor genes (**Figure 9**). Briefly, a gene copy is transcribed into a primary RNA sequence that is spliced to remove introns during the production of multiple mature RNAs. The number of introns in a mature RNA varies. The generated RNA sequences are then reverse-transcribed into DNA fragments that are subsequently inserted into new chromosomal locations. New genes duplicate over time, producing subfamilies comprising variable gene copies. Because they were derived from the same progenitor gene, they often have related biochemical functions. This model is supported by the findings of previous studies (Brosius, 1991; Wang et al., 2006; Bai et al., 2008; Kaessmann et al., 2009; Panchy et al., 2016).

## 4 Conclusion

The origin of IGs may be mediated by ancient RNA splicing, reverse transcription, and recombination. The typical features of the rapid evolution of IGs include the following: recent duplication event, variable copy numbers, low divergence between paralogs within a genome, and high non-synonymous-to-synonymous substitution ratios. A genome-wide analysis of IG families and their phylogenetic relationships revealed the evolutionary dynamics of IGs in various *Poaceae* subfamilies. Under relaxed selection pressure, retrotransposition, intron loss, and gene duplication and conversion may accelerate the expansion of IG families. Our findings provide critical insights into the evolutionary trajectory of IGs in *Poaceae*.

## 5 Materials and methods

### 5.1 Sequence data

Annotated genome sequences were downloaded from public databases, including Ensembl (http://plants.ensembl.org/) and Phytozome (http://www.phytozome.net/). The following nine plants were selected as representative *Poaceae* species: *B. distachyon* (2n = 10) (Fox et al., 2013), *E. curvula* (2n = 20) (Carballo et al., 2019), *L. perrieri* (2n = 24) (Loera-Sánchez et al., 2022), *O. sativa* (2n = 24) (Kawahara et al., 2013), *P. hallii* (2n = 18) (Lovell et al., 2018), *S. italica* (2n = 18) (Bennetzen et al., 2012), *S. bicolor* (2n = 20) (Paterson et al., 2009), *Z. mays* (2n = 20) (Hufford et al., 2021), and *O. thomaeum* (2n = 18) (VanBuren et al., 2015).

### 5.2 Data extraction and processing of intronless genes and multiexon genes

The IGs in the nine species were obtained using the General Feature Format Version 3 (GFF3) file. First, genes that contain the line “CDS” were extracted from the GFF3 file. Redundant sequences representing the same loci were excluded. Genes containing only one line for “exon” were extracted from each genome and used as candidate sequences for further analyses. If there was only one line for “exons,” the coding sequence was considered to lack introns and the gene was designated as intronless. Because mitochondrial and chloroplast DNA do not contain introns, genes labeled “MT” and “PT” were deleted. Genes that were not mapped to chromosomes were also eliminated. To ensure that IGs were accurately identified, all candidate genes were verified using the SMART online tool (http://smart.embl-heidelberg.de). Finally, a non-redundant IG data set for nine *Poaceae* species was generated. After excluding the IGs, the remaining genes were considered to be potential MEGs. The longest coding sequences were selected as the representative transcripts to generate MEG data sets for the subsequent analyses. The number of introns in each coding gene was extracted from the GFF3 file using the Python script (https://github.com/irusri/Extract-intron-from-gff3).

### 5.3 Identification of paralogy

We aligned the coding sequences of the IGs to their paralogs using the following parameters of GAMP: –min-intronlength 9 -z sense_force –min-trimmed-coverage 0.7 –min-identity 0.7 (Wu and Watanabe, 2005). For each species, we built a GFF3 file containing information regarding the homology between IGs and MEGs (**Supplementary Table S1**).

### 5.4 Analysis of gene family expansion and contraction

The *Arabidopsis thaliana*, *Cinnamomum chinensis*, and *Amborella trichopoda* genes were selected as outgroups. The homologous groups of the nine species were clustered on the basis of protein sequences using OrthoFinder (Emms and Kelly, 2015). The phylogenetic tree for the selected species was reconstructed using RAxML (Stamatakis, 2014) according to the optimal amino acid model (JTT+I+G+F) inferred by ProtTest (Posada, 2011) for the 242 single-copy homologous gene families generated by the cluster analysis of homologous proteins. The CAFE software was used to detect significant gene family expansions and contractions (*P* = 0.05) (Bie et al., 2006). Additionally, R8s was used to calibrate the divergence time (Taylor and Berbee, 2006) according to the following constraints: (1) the divergence time for *A. thaliana* and *O. sativa* was 152 Mya (Magallóan and Sanderson, 2010), and (2) the divergence time for *Z. mays* and *S. italica* was 23.4 Mya (Pessoa-Filho et al., 2017). An orthogroup with 50%–70% of its genes lacking introns was inferred to be an orthogroup of IGs. A total of 548 IGs were screened from homologous groups using this criterion. The branch-specific intron gain and loss rates were calculated using the default parameters of the Malin software (Csűrös, 2008). The intron sites evolved independently according to the Markov model (Steel, 1994).

### 5.5 Analysis of gene duplications and selection pressures

Different gene duplication patterns were identified using DupGen_finder (Qiao et al., 2019). First, an all-vs.-all local BLASTP search was performed using protein sequences to screen for potential homologous gene pairs in each genome (E < 1e-10, top 5 matches, and m8 format output). Then, the *A. thaliana* protein sequence was selected as the outgroup and the DupGen_finder-unique program was used to identify five gene duplication events (i.e., TD, PD, DSD, TRD, and WGD). The detect_gene_conversion pipeline (Qiao et al., 2019) was used to identify homologous gene quartets and analyze gene conversions.

The synonymous substitution rate (Ks) and the non-synonymous substitution rate (Ka) for the duplicated gene pairs were calculated using KaKs_calculator 2.0 and the NG model (Wang et al., 2010) (**Supplementary Table S3**). The Ks values were converted to divergence times using the formula T = Ks·(2r)^−1^, where T is the divergence time and r is the neutral substitution rate (6.50 × 10^−9^) (Gaut et al., 1996).

### 5.6 Analysis of functional enrichment and gene expression

Functional enrichment was assessed *via* an over-representation analysis. All enrichment analyses were performed using a hypergeometric test (*P* < 0.05). The default parameters of the ClusterProfiler R package were applied to analyze and visualize data (Yu et al., 2012). Rice and maize tissue-specific expression data were obtained from the Expression Atlas platform (https://www.ebi.ac.uk/gxa/home).

### 5.7 Examination of retrotransposon density

A retrotransposon model file was obtained from the Dfam database (https://www.dfam.org/home). On the basis of the similarity search algorithm of the Hidden Markov model, the nucleotide sequences (6 kb) upstream and downstream of genes were examined for the presence of Copia and Gypsy retrotransposons using the nhmmscan program of HMMER (E < 1e-10) (**Supplementary Table S2**). The number of retrotransposons was determined for 100-bp intervals from the transcription start site to 4 kb upstream and from the transcription termination site to 4 kb downstream. The data were normalized according to the number of IGs or MEGs.

## Data availability statement

The original contributions presented in the study are included in the article/**Supplementary Material**. Further inquiries can be directed to the corresponding authors.

## Author contributions

YC and LM conceived and designed the study. LM and TZ provided administrative support. YC and TM collected, analyzed, and interpreted the data. LM and TZ wrote the manuscript. All authors reviewed the manuscript and approved the submitted version.

## References

[B1] Adams K. L. Wendel J. F. (2005). Polyploidy and genome evolution in plants. Curr. Opin. Plant Biol. 8 (2), 135–141. doi: 10.1016/j.pbi.2005.01.001 15752992

[B2] Bai Y. Casola C. Betrán E. (2008). Evolutionary origin of regulatory regions of retrogenes in drosophila. BMC Genomics 9, 241. doi: 10.1186/1471-2164-9-241 18498650 PMC2413143

[B3] Barker M. S. Li Z. Kidder T. I. Reardon C. R. Lai Z. Oliveira L. O.. (2016). Most compositae (Asteraceae) are descendants of a paleohexaploid and all share a paleotetraploid ancestor with the calyceraceae. Am. J. Bot. 103 (7), 1203–1211. doi: 10.3732/ajb.1600113 27313199

[B4] Bennetzen J. L. Schmutz J. Wang H. Percifield R. Hawkins J. Pontaroli A. C.. (2012). Reference genome sequence of the model plant setaria. Nat. Biotechnol. 30 (6), 555–561. doi: 10.1038/nbt.2196 22580951

[B5] Bie T. D. Cristianini N. Demuth J. P. Hahn W. ,. M. (2006). CAFE: a computational tool for the study of gene family evolution. Bioinformatics 22 (10), 1269–1271. doi: 10.1093/bioinformatics/btl097 16543274

[B6] Bowers J. E. Chapman B. A. Rong J. Paterson A. H. (2003). Unravelling angiosperm genome evolution by phylogenetic analysis of chromosomal duplication events. Nature 422 (6930), 433–438. doi: 10.1038/nature01521 12660784

[B7] Brosius J. (1991). Retroposons–seeds of evolution. Science 251 (4995), 753. doi: 10.1126/science.1990437 1990437

[B8] Carballo J. Santos B. Zappacosta D. Garbus I. Selva J. P. Gallo C. A.. (2019). A high-quality genome of eragrostis curvula grass provides insights into poaceae evolution and supports new strategies to enhance forage quality. Sci. Rep. 9 (1), 10250. doi: 10.1038/s41598-019-46610-0 31308395 PMC6629639

[B9] Carmel L. Wolf Y. I. Rogozin I. B. Koonin E. V. (2007). Three distinct modes of intron dynamics in the evolution of eukaryotes. Genome Res. 17 (7), 1034–1044. doi: 10.1101/gr.6438607 17495008 PMC1899114

[B10] Carvunis A. R. Rolland T. Wapinski I. Calderwood M. A. Yildirim M. A. Simonis N.. (2012). Proto-genes and *de novo* gene birth. Nature 487 (7407), 370–374. doi: 10.1038/nature11184 22722833 PMC3401362

[B11] Cazorla O. Freiburg A. Helmes M. Centner T. McNabb M. Wu Y.. (2000). Differential expression of cardiac titin isoforms and modulation of cellular stiffness. Circ. Res. 86 (1), 59–67. doi: 10.1161/01.res.86.1.59 10625306

[B12] Chen L. Zhao J. Song J. Jameson P. E. (2021). Cytokinin glucosyl transferases, key regulators of cytokinin homeostasis, have potential value for wheat improvement. Plant Biotechnol. J. 19 (5), 878–896. doi: 10.1111/pbi.13595 33811433 PMC8131048

[B13] Cheng F. Wu J. Cai X. Liang J. Freeling M. Wang X. (2018). Gene retention, fractionation and subgenome differences in polyploid plants. Nat. Plants 4 (5), 258–268. doi: 10.1038/s41477-018-0136-7 29725103

[B14] Chorev M. Carmel L. (2012). The function of introns. Front. Genet. 3. doi: 10.3389/fgene.2012.00055 PMC3325483 22518112

[B15] Clark J. W. Donoghue P. (2018). Whole-genome duplication and plant macroevolution. Trends Plant ence 23, 933–945. doi: 10.1016/j.tplants.2018.07.006 30122372

[B16] Cohen N. E. Shen R. Carmel L. (2012). The role of reverse transcriptase in intron gain and loss mechanisms. Mol. Biol. Evol. 29 (1), 179–186. doi: 10.1093/molbev/msr192 21804076

[B17] Csűrös M. (2008). Malin: Maximum likelihood analysis of intron evolution in eukaryotes. Bioinformatics 24 (13), 1538–1539. doi: 10.1093/bioinformatics/btn226 18474506 PMC2718671

[B18] Csűrös M. Holey J. A. Rogozin I. B. (2007). In search of lost introns. Bioinformatics 23 (13), i87–i96. doi: 10.1093/bioinformatics/btm190 17646350

[B19] Csűrös M. Rogozin I. B. Koonin E. V. (2011). A detailed history of intron-rich eukaryotic ancestors inferred from a global survey of 100 complete genomes. PLos Comput. Biol. 7 (9), e1002150. doi: 10.1371/journal.pcbi.1002150 21935348 PMC3174169

[B20] Emms D. M. Kelly S. (2015). OrthoFinder: Solving fundamental biases in whole genome comparisons dramatically improves orthogroup inference accuracy. Genome Biol. 16 (157), 157. 26243257 10.1186/s13059-015-0721-2 PMC4531804

[B21] Esnault C. Maestre J. Heidmann T. (2000). Human LINE retrotransposons generate processed pseudogenes. Nat. Genet. 24 (4), 363–367. doi: 10.1038/74184 10742098

[B22] Felsenstein J. (1978). Cases in which parsimony and compatibility will be positively misleading. Systematic Zoology 27 (4), 401–410. doi: 10.1093/sysbio/27.4.401

[B23] Fox S. E. Preece J. Kimbrel J. A. Marchini G. L. Sage A. Youens-Clark K.. (2013). Sequencing and *de novo* transcriptome assembly of brachypodium sylvaticum (Poaceae). Appl. Plant Sci. 1 (3), 1200011. doi: 10.3732/apps.1200011 PMC4105277 25202520

[B24] Gaut B. S. Morton B. R. Mccaig B. C. (1996). Substitution rate comparisons between grasses and palms: Synonymous rate differences at the nuclear gene adh parallel rate differences at the plastid gene rbcL. Proc. Natl. Acad. Sci. United States America 93 (19), p.10274–10279. doi: 10.1073/pnas.93.19.10274 PMC38374 8816790

[B25] Gentles A. J. Karlin S. (1999). Why are human G-protein-coupled receptors predominantly intronless? Trends Genet. 15 (2), 47–49. doi: 10.1016/s0168-9525(98)01648-5 10098406

[B26] Grau-Bové X. Torruella G. Donachie S. Suga H. Leonard G. Richards T. A.. (2017). Dynamics of genomic innovation in the unicellular ancestry of animals. Elife 6 (20), e26036. doi: 10.7554/eLife.26036 28726632 PMC5560861

[B27] Holland P. W. H. Marlétaz F. Maeso I. Dunwell T. L. Paps J. (2017). New genes from old: Asymmetric divergence of gene duplicates and the evolution of development. Philos. Trans. R. Soc. B Biol. Sci. 372 (1713), 20150480. doi: 10.1098/rstb.2015.0480 PMC5182412 27994121

[B28] Hufford M. B. Seetharam A. S. Woodhouse M. R. Chougule K. M. Ou S. Liu J.. (2021). *De novo* assembly, annotation, and comparative analysis of 26 diverse maize genomes. Science 373 (6555), 655–662. doi: 10.1126/science.abg5289 34353948 PMC8733867

[B29] Jain M. Khurana P. Tyagi A. K. Khurana J. P. (2008). Genome-wide analysis of intronless genes in rice and arabidopsis. Funct. Integr. Genomics 8 (1), 69–78. doi: 10.1007/s10142-007-0052-9 17578610

[B30] Jiao Y. Li J. Tang H. Paterson A. H. (2014). Integrated syntenic and phylogenomic analyses reveal an ancient genome duplication in monocots. Plant Cell 26 (7), 2792–2802. doi: 10.1105/tpc.114.127597 25082857 PMC4145114

[B31] Jiao Y. Paterson A. H. (2014). Polyploidy-associated genome modifications during land plant evolution. Philos. Trans. R Soc. Lond B Biol. Sci. 369 (1648), 20130355. doi: 10.1098/rstb.2013.0355 24958928 PMC4071528

[B32] Kaessmann H. Vinckenbosch N. Long M. (2009). RNA-Based gene duplication: Mechanistic and evolutionary insights. Nat. Rev. Genet. 10 (1), 19–31. doi: 10.1038/nrg2487 19030023 PMC3690669

[B33] Kawahara Y. de la Bastide M. Hamilton J. P. Kanamori H. McCombie W. R. Ouyang S.. (2013). Improvement of the oryza sativa nipponbare reference genome using next generation sequence and optical map data. Rice (N Y) 6 (1), 4. doi: 10.1186/1939-8433-6-4 24280374 PMC5395016

[B34] Knowles D. G. McLysaght A. (2006). High rate of recent intron gain and loss in simultaneously duplicated arabidopsis genes. Mol. Biol. Evol. 23 (8), 1548–1557. doi: 10.1093/molbev/msl017 16720694

[B35] Liu J. Chen N. Chen F. Cai B. Dal Santo S. Tornielli G. B.. (2014). Genome-wide analysis and expression profile of the bZIP transcription factor gene family in grapevine (Vitis vinifera). BMC Genomics 15, 281. doi: 10.1186/1471-2164-15-281 24725365 PMC4023599

[B36] Liu H. Lyu H. M. Zhu K. Van de Peer Y. Max Cheng Z. M. (2021). The emergence and evolution of intron-poor and intronless genes in intron-rich plant gene families. Plant J. 105 (4), 1072–1082. doi: 10.1111/tpj.15088 33217085 PMC7116809

[B37] Loera-Sánchez M. Studer B. Kölliker R. (2022). A multispecies amplicon sequencing approach for genetic diversity assessments in grassland plant species. Mol. Ecol. Resour 22 (5), 1725–1745. doi: 10.1111/1755-0998.13577 34918474 PMC9305562

[B38] Louhichi A. Fourati A. Rebaï A. (2011). IGD: a resource for intronless genes in the human genome. Gene 488 (1-2), 35–40. doi: 10.1016/j.gene.2011.08.013 21914464

[B39] Lovell J. T. Jenkins J. Lowry D. B. Mamidi S. Sreedasyam A. Weng X.. (2018). The genomic landscape of molecular responses to natural drought stress in panicum hallii. Nat. Commun. 9 (1), 5213 doi: 10.1038/s41467-018-07669-x 30523281 PMC6283873

[B40] Lynch M. Conery J. S. (2000). The evolutionary fate and consequences of duplicate genes. Science 290 (5494), 1151–1155. doi: 10.1126/science.290.5494.1151 11073452

[B41] Magallóan S. A. Sanderson M. J. (2010). Angiosperm divergence times: The effect of genes, codon positions, and time constraints. Evolution 59 (8), 1653–1670. doi: 10.1554/04-565.1 16329238

[B42] Mondragon-Palomino M. Gaut B. S. (2005). Gene conversion and the evolution of three leucine-rich repeat gene families in arabidopsis thaliana. Mol. Biol. Evol. 22 (12), 2444–2456. doi: 10.1093/molbev/msi241 16120808

[B43] Panchy N. Lehti-Shiu M. Shiu S. H. (2016). Evolution of gene duplication in plants. Plant Physiol. 171 (4), 2294–2316. doi: 10.1104/pp.16.00523 27288366 PMC4972278

[B44] Paterson A. H. Bowers J. E. Bruggmann R. Dubchak I. Grimwood J. Gundlach H.. (2009). The sorghum bicolor genome and the diversification of grasses. Nature 457 (7229), 551–556. doi: 10.1038/nature07723 19189423

[B45] Pessoa-Filho M. Martins A. M. ME F. (2017). Molecular dating of phylogenetic divergence between urochloa species based on complete chloroplast genomes. BMC Genomics 18 (1), 516. doi: 10.1186/s12864-017-3904-2 28683832 PMC5499013

[B46] Posada D. (2011). ProtTest 3: fast selection of best-fit models of protein evolution. Bioinformatics 27 (8), 1164–1165. doi: 10.1093/bioinformatics/btr088 21335321 PMC5215816

[B47] Qiao X. Li Q. Yin H. Qi K. Li L. Wang R.. (2019). Gene duplication and evolution in recurring polyploidization–diploidization cycles in plants. Genome Biol. 20 (1), 1–23. doi: 10.1186/s13059-019-1650-2 30791939 PMC6383267

[B48] Roy S. W. Penny D. (2007). Patterns of intron loss and gain in plants: Intron loss-dominated evolution and genome-wide comparison of o. sativa and a. thaliana. Mol. Biol. Evol. 24 (1), 171–1781. doi: 10.1093/molbev/msl159 17065597

[B49] Sakharkar M. K. Chow V. T. Ghosh K. Chaturvedi I. Lee P. C. Bagavathi S. P.. (2005). Computational prediction of SEG (single exon gene) function in humans. Front. Biosci. 10, 1382–1395. doi: 10.2741/1627 15769633

[B50] Savisaar R. Hurst L. D. (2016). Purifying selection on exonic splice enhancers in intronless genes. Mol. Biol. Evol. 33 (6), 1396–1418. doi: 10.1093/molbev/msw018 26802218 PMC4868121

[B51] Sawyer S. (1989). Statistical tests for detecting gene conversion. Mol. Biol. Evol. 6 (5), 526–538. doi: 10.1093/oxfordjournals.molbev.a040567 2677599

[B52] Smith S. A. Brown J. W. Yang Y. Bruenn R. Moore M. J. (2017). Disparity, diversity, and duplications in the caryophyllales. New Phytol. 217 (2), 836–854. doi: 10.1111/nph.14772 28892163

[B53] Soreng R. Peterson P. Romaschenko K. Davidse G. Teisher J. Clark L.. (2017). A worldwide phylogenetic classification of the poaceae (Gramineae) II: An update and a comparison of two 2015 classifications: Soreng et al.: Phylogenetic classification of the grasses II. J. Systematics Evol. 55, 259–290. doi: 10.1111/jse.12262

[B54] Soreng R. J. Peterson P. M. Romaschenko K. Davidse G. Zuloaga F. O. Judziewicz E. J.. (2015). A worldwide phylogenetic classification of the poaceae (Gramineae), Vol. 53 (2). 117–137. doi: 10.1111/jse.12150

[B55] Soreng R. J. Peterson P. M. Zuloaga F. O. Romaschenko K. Clark L. G. Teisher J. K.. (2022). A worldwide phylogenetic classification of the poaceae (Gramineae) III. J. Systematics Evol. 60 (3), 476–521. doi: 10.1111/jse.12847

[B56] Souza S. (2003). The emergence of a synthetic theory of intron evolution. Genetica 118 (2-3), 117–121. doi: 10.1007/978-94-010-0229-5_2 12868602

[B57] Spellman R. Llorian M. Smith C. W. (2007). Crossregulation and functional redundancy between the splicing regulator PTB and its paralogs nPTB and ROD1. Mol. Cell 27 (3), 420–434. doi: 10.1016/j.molcel.2007.06.016 17679092 PMC1940037

[B58] Stajich J. E. Dietrich F. S. (2006). Evidence of mRNA-mediated intron loss in the human-pathogenic fungus cryptococcus neoformans. Eukaryot Cell 5 (5), 789–793. doi: 10.1128/ec.5.5.789-793.2006 16682456 PMC1459680

[B59] Stamatakis A. (2014). RAxML version 8: a tool for phylogenetic analysis and post-analysis of large phylogenies. Bioinformatics 30 (9), 1312–1313. doi: 10.1093/bioinformatics/btu033 24451623 PMC3998144

[B60] Stanley K. E. (1999). Evolutionary trends in the grasses (Poaceae): A review. Michigan Botanist 38 (1), 3. doi: 10.1038/ng940

[B61] Steel M. (1994). Recovering a tree from the leaf colourations it generates under a Markov model. Appl. Mathematics Lett. 7 (2), 19–23. doi: 10.1016/0893-9659(94)90024-8

[B62] Taylor J. W. Berbee M. L. (2006). Dating divergences in the fungal tree of life: review and new analyses. Mycologia 98 (6), 838–849. doi: 10.3852/mycologia.98.6.838 17486961

[B63] Thomasson J. R. (1980). Paleoagrostology: A historical review. Iowa State J. Res. 54, 301–317.

[B64] VanBuren R. Bryant D. Edger P. P. Tang H. Burgess D. Challabathula D.. (2015). Single-molecule sequencing of the desiccation-tolerant grass oropetium thomaeum. Nature 527 (7579), 508–511. doi: 10.1038/nature15714 26560029

[B65] Wang X. Tang H. Bowers J. E. Feltus F. A. Paterson A. H. (2007). Extensive concerted evolution of rice paralogs and the road to regaining independence. Genetics 177 (3), 1753–1763. doi: 10.1534/genetics.107.073197 18039882 PMC2147972

[B66] Wang. Y. P. Wang X. Paterson A. H. (2012). Genome and gene duplications and gene expression divergence: A view from plants. Ann. NY Acad. Sci. 1256 (-), 1–14. doi: 10.1111/j.1749-6632.2011.06384.x 22257007

[B67] Wang D. Zhang Y. Zhang Z. Zhu J. Yu J. (2010). KaKs_Calculator 2.0: A toolkit incorporating gamma-series methods and sliding window strategies. Genomics Proteomics Bioinf. 8 (1), 77–80. doi: 10.1016/S1672-0229(10)60008-3 PMC5054116 20451164

[B68] Wang W. Zheng H. Fan C. Li J. Shi J. Cai Z.. (2006). High rate of chimeric gene origination by retroposition in plant genomes. Plant Cell 18 (8), 1791–1802. doi: 10.1105/tpc.106.041905 16829590 PMC1533979

[B69] Weisman C. M. Murray A. W. Eddy S. R. (2020). Many, but not all, lineage-specific genes can be explained by homology detection failure. PLos Biol. 18 (11), e3000862. doi: 10.1371/journal.pbio.3000862 33137085 PMC7660931

[B70] Wendel J. F. Jackson S. A. Meyers B. C. Wing R. A. (2016). Evolution of plant genome architecture. Genome Biol. 17, 37. doi: 10.1186/s13059-016-0908-1 26926526 PMC4772531

[B71] Werner M. S. Sieriebriennikov B. Prabh N. Loschko T. Lanz C. Sommer R. J. (2018). Young genes have distinct gene structure, epigenetic profiles, and transcriptional regulation. Genome Res. 28 (11), 1675–1687. doi: 10.1101/gr.234872.118 30232198 PMC6211652

[B72] White M. E. Crother B. I. (2000). Gene conversions may obscure actin gene family relationships. J. Mol. Evol. 50 (2), 170–174. doi: 10.1007/s002399910018 10684350

[B73] Wolfe C. K. H. (2007). Not born equal: Increased rate asymmetry in relocated and retrotransposed rodent gene duplicates. Mol. Biology&Evolution 24 (3), 679–686. doi: 10.1093/molbev/msl199 17179139

[B74] Worden A. Z. Lee J. H. Mock T. Rouzé P. Simmons M. P. Aerts A. L.. (2009). Green evolution and dynamic adaptations revealed by genomes of the marine picoeukaryotes micromonas. Science 324 (5924), 268–272. doi: 10.1126/science.1167222 19359590

[B75] Wu T. D. Watanabe C. K. (2005). GMAP: a genomic mapping and alignment program for mRNA and EST sequences. Bioinformatics 21 (9), 1859–1875. doi: 10.1093/bioinformatics/bti310 15728110

[B76] Yan H. Zhang W. Lin Y. Dong Q. Peng X. Jiang H.. (2014). Different evolutionary patterns among intronless genes in maize genome. Biochem. Biophys. Res. Commun. 449 (1), 146–150. doi: 10.1016/j.bbrc.2014.05.008 24820954

[B77] Yu G. Wang L. G. Han Y. He Q. Y. (2012). clusterProfiler: An r package for comparing biological themes among gene clusters. Omics-a J. Integr. Biol. 16 (5), 284–287. doi: 10.1089/omi.2011.0118 PMC3339379 22455463

[B78] Zhang X. Firestein S. (2002). The olfactory receptor gene superfamily of the mouse. Nat. Neurosci. 5 (2), 124–133. doi: 10.1038/nn800 11802173

[B79] Zhong Y. Zhang X. Cheng Z. M. (2018). Lineage-specific duplications of NBS-LRR genes occurring before the divergence of six fragaria species. BMC Genomics 19 (1), 128. doi: 10.1186/s12864-018-4521-4 29422035 PMC5806312

[B80] Zhu K. Wang X. Liu J. Tang J. Cheng Q. Chen J. G.. (2018). The grapevine kinome: annotation, classification and expression patterns in developmental processes and stress responses. Hortic. Res. 5, 19. doi: 10.1038/s41438-018-0027-0 29619230 PMC5878832

