# Supplementary material for: Trends in the evolution of intronless genes in Poaceae
Source: Front Plant Sci. 2023 Feb 16;14:1065631. doi: 10.3389/fpls.2023.1065631 (PMC9978806; doi:10.3389/fpls.2023.1065631)
Supplement: Supplementary file 5 [file Presentation_1.pdf]

Supplementary Material

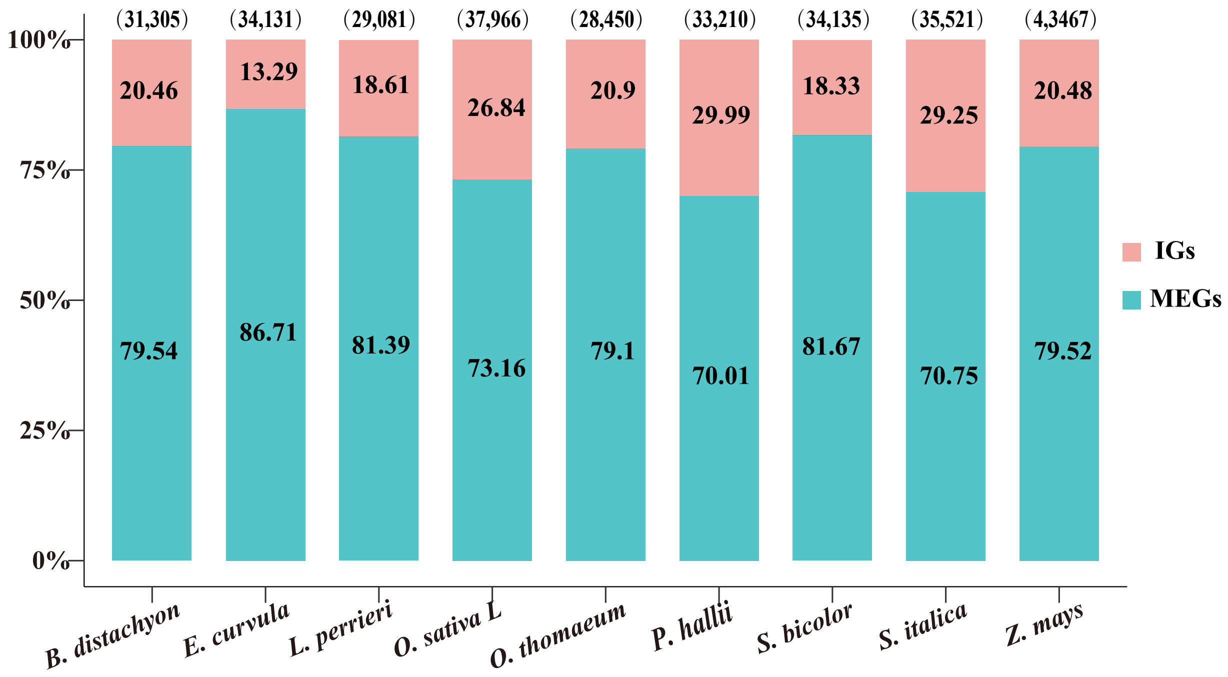

Supplementary Figure S1. Percentages of intronless genes (IGs) and multi-exon genes (MEGs) in the nine examined species. The number of genes is shown in parentheses above each bar.

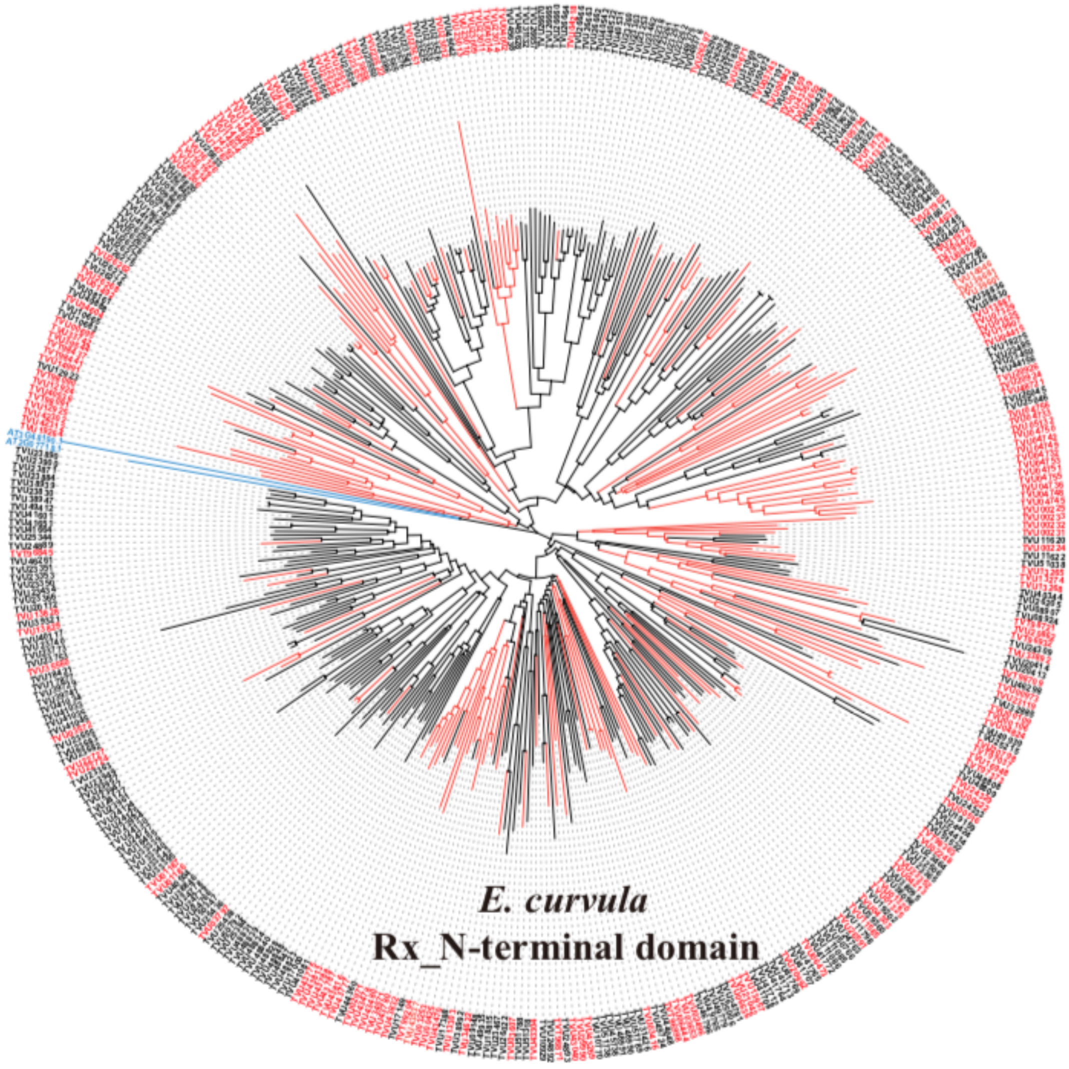
E. curvula
Rx_N-terminal domain

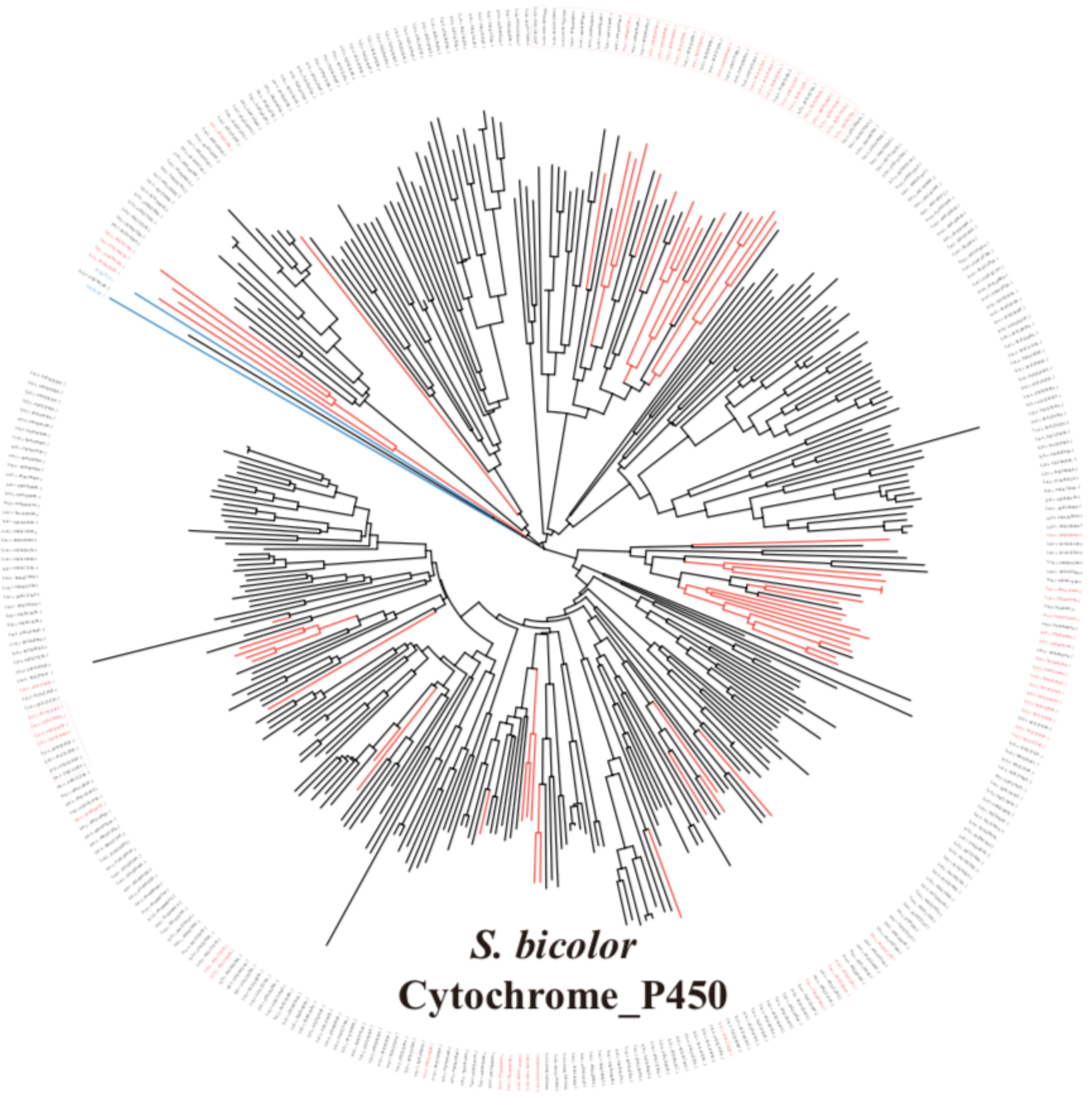

Supplementary Figure S2. Gene family tree for the Rx_N-terminal domain in *E. curvalua* and Cytochrome_P450 in *S. bicolor.* Red, black, and blue branches with node labels represent intronless genes (IGs), multi-exon genes (MEGs), and outgroups (*Cinnamomum chinensis* and *Arabidopsis thaliana*), respectively.

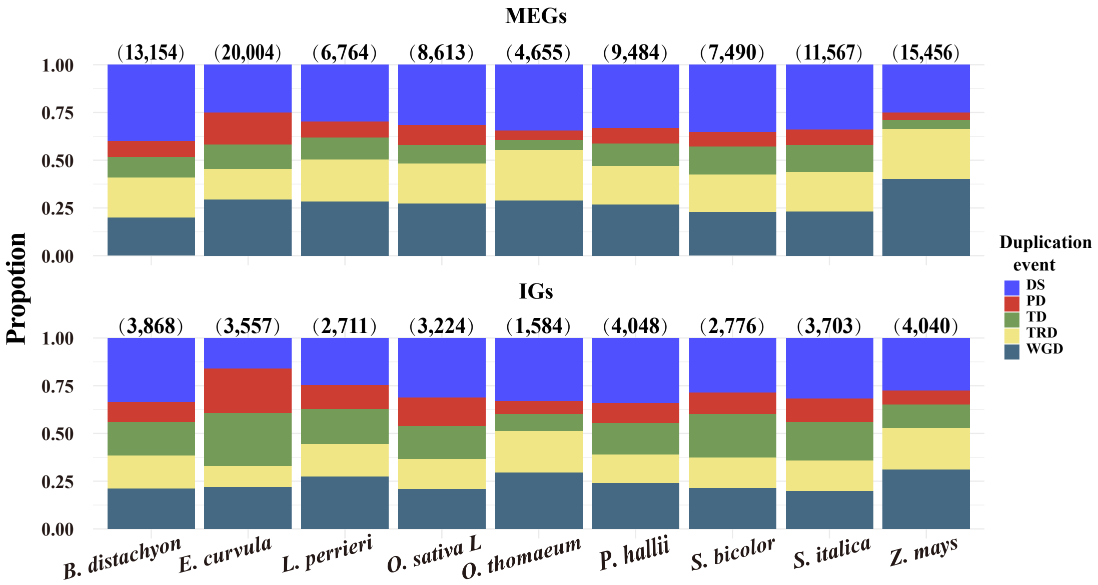

Supplementary Figure S3. Proportions of duplicated intronless genes (IGs) and multi-exon genes (MEGs) assigned to duplication event categories in nine genomes. Five gene duplication events are presented: tandem (TD), proximal (PD), dispersed (DSD), transposed (TRD), and whole-genome (WGD) duplications.

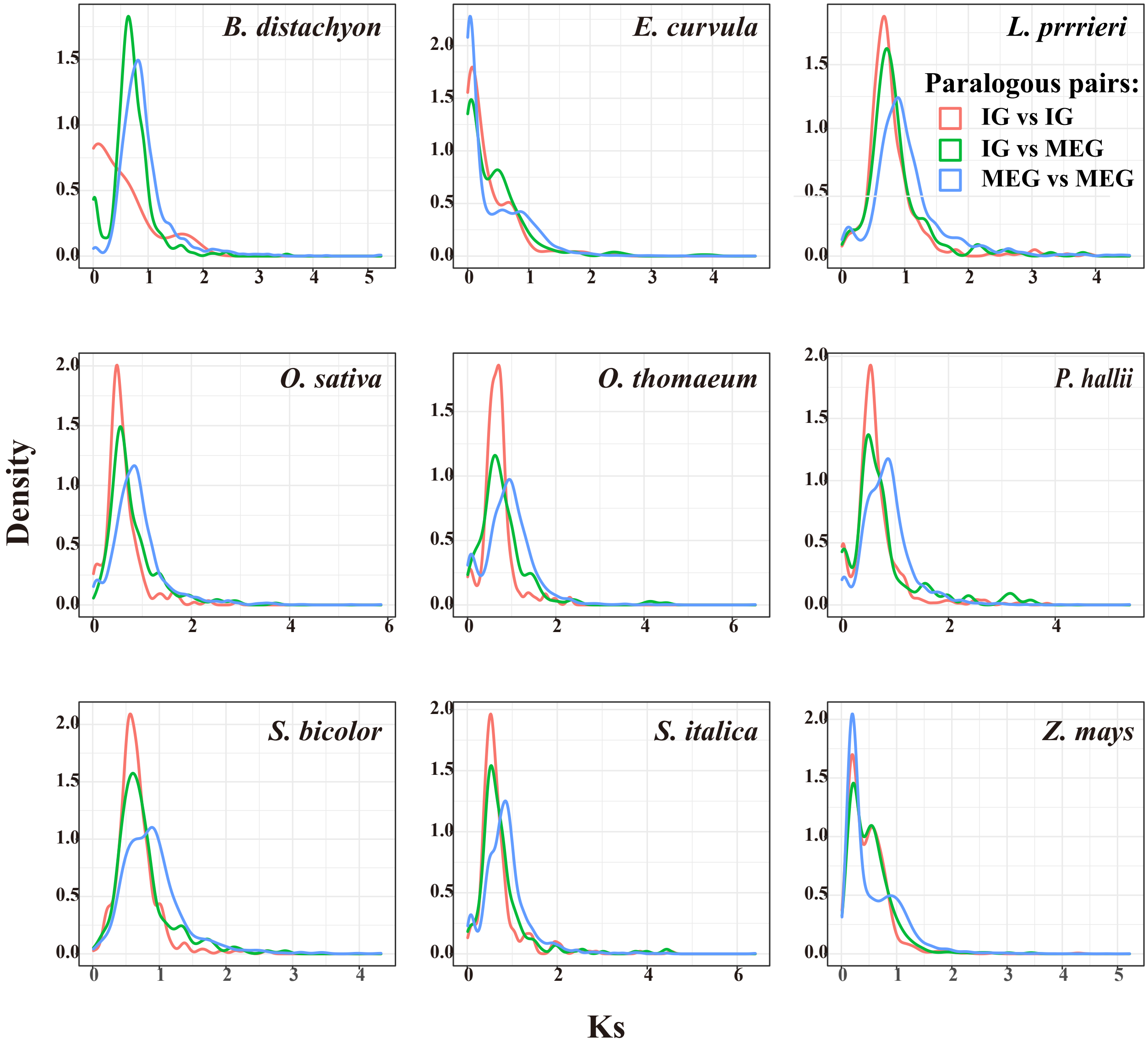

Supplementary Figure S4. Distribution of Ks values. Density plot of the Ks values for three paralogous sets: intron-free (IG vs IG), transition (IG vs MEG), and intron-rich (MEG vs MEG). IG, intronless gene; MEG, multi-exon gene.

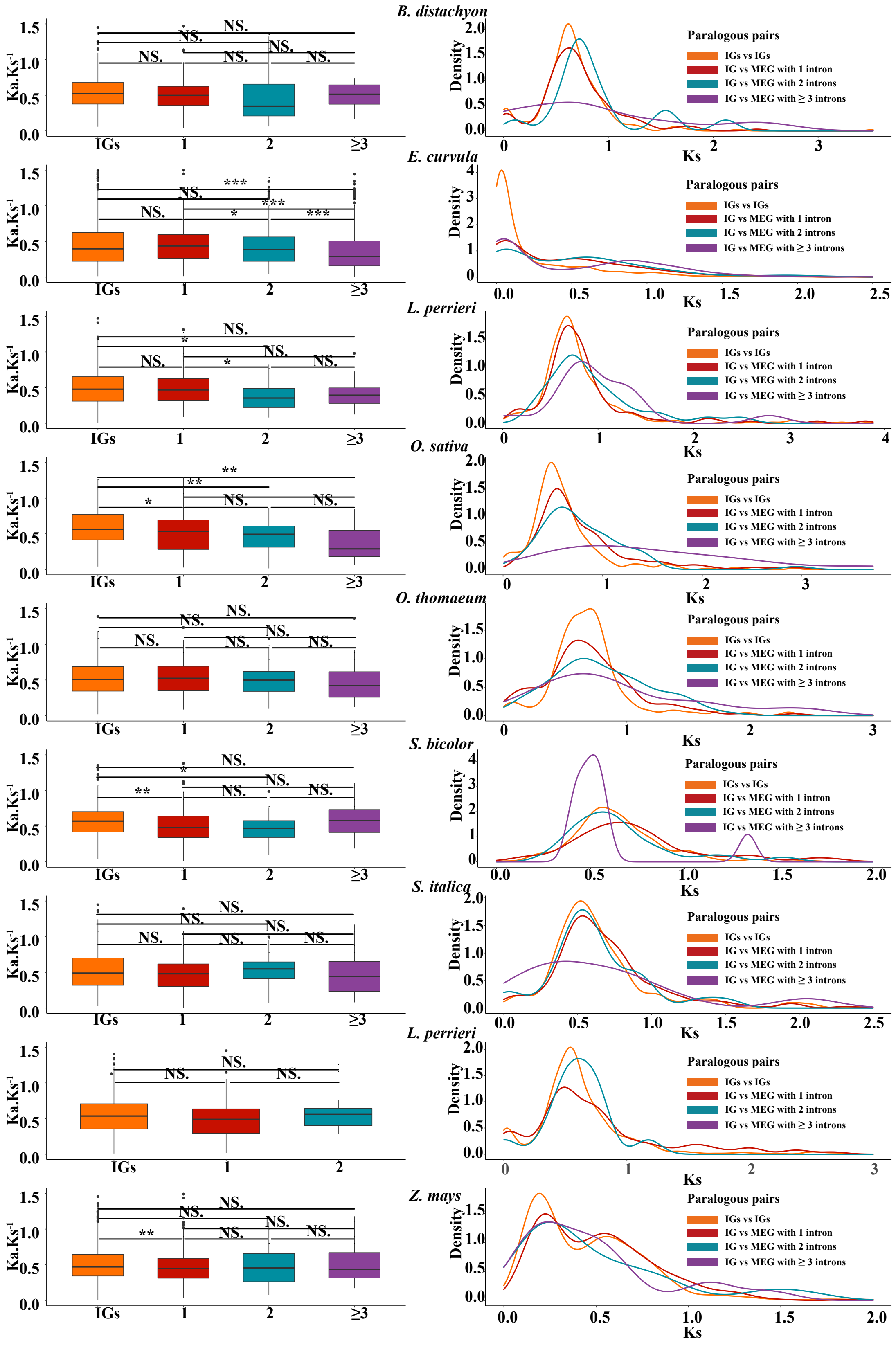

B. distachyon
E. curvula
L. perrieri
O. sativa
O. thomaeum
S. bicolor
S. italica
L. perrieri
Z. mays
Ka.Ks-1
IGs
1
2
≥3
NS.
*
**
***
Density
Ks
Paralogous pairs
IGs vs IGs
IG vs MEG with 1 intron
IG vs MEG with 2 introns
IG vs MEG with ≥ 3 introns

Supplementary Figure S5. Distribution of Ks and $Ka \cdot Ks^{-1}$ values for paralogous pairs between intronless genes (IGs) and multi-exon genes (MEGs) with one, two, and three or more intron(s). The boxplots present the mean, upper and lower quartiles, and 95% confidence intervals for $Ka \cdot Ks^{-1}$. Points indicate outliers in the data. Asterisks represent significant differences (two-sided paired Wilcoxon sign test: *$P < 0.05$, **$P < 0.01$, and ***$P < 0.001$).

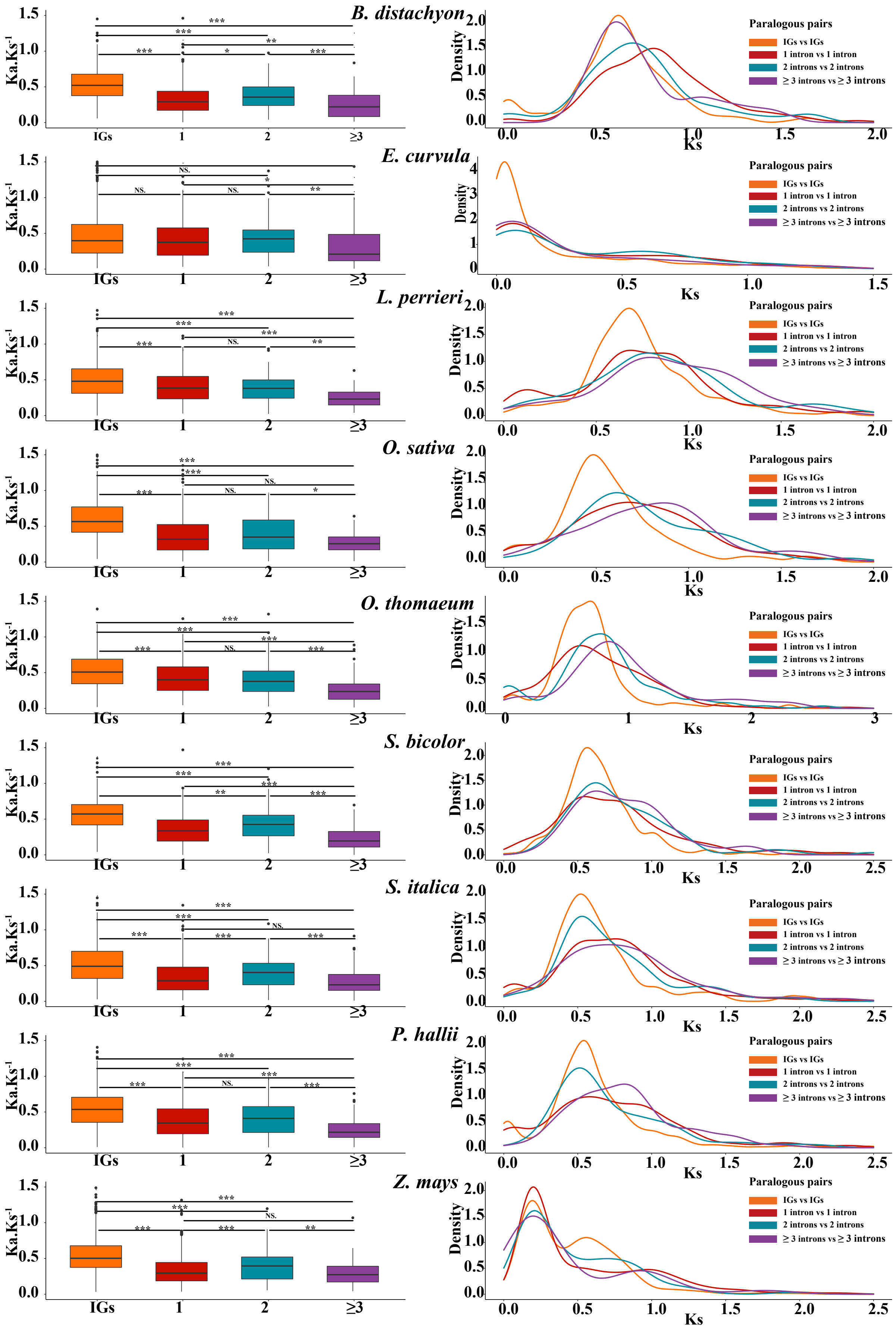
B. distachyon
E. curvula
L. perrieri
O. sativa
O. thomaeum
S. bicolor
S. italica
P. hallii
Z. mays
Ka.Ks-1
IGs
1
2
≥3
***
**
*
NS.
Density
Dnsity
Ks
Paralogous pairs
IGs vs IGs
1 intron vs 1 intron
2 introns vs 2 introns
≥ 3 introns vs ≥ 3 introns

Supplementary Figure S6. Distribution of Ks and Ka·Ks^{-1} values for paralogous pairs of genes with the same number of introns. The boxplots present the mean, upper and lower quartiles, and 95% confidence intervals for Ka·Ks^{-1}. Points indicate outliers in the data. Asterisks represent significant differences (two-sided paired Wilcoxon sign test: *$P < 0.05$, **$P < 0.01$, and ***$P < 0.001$). IGs, intronless genes.

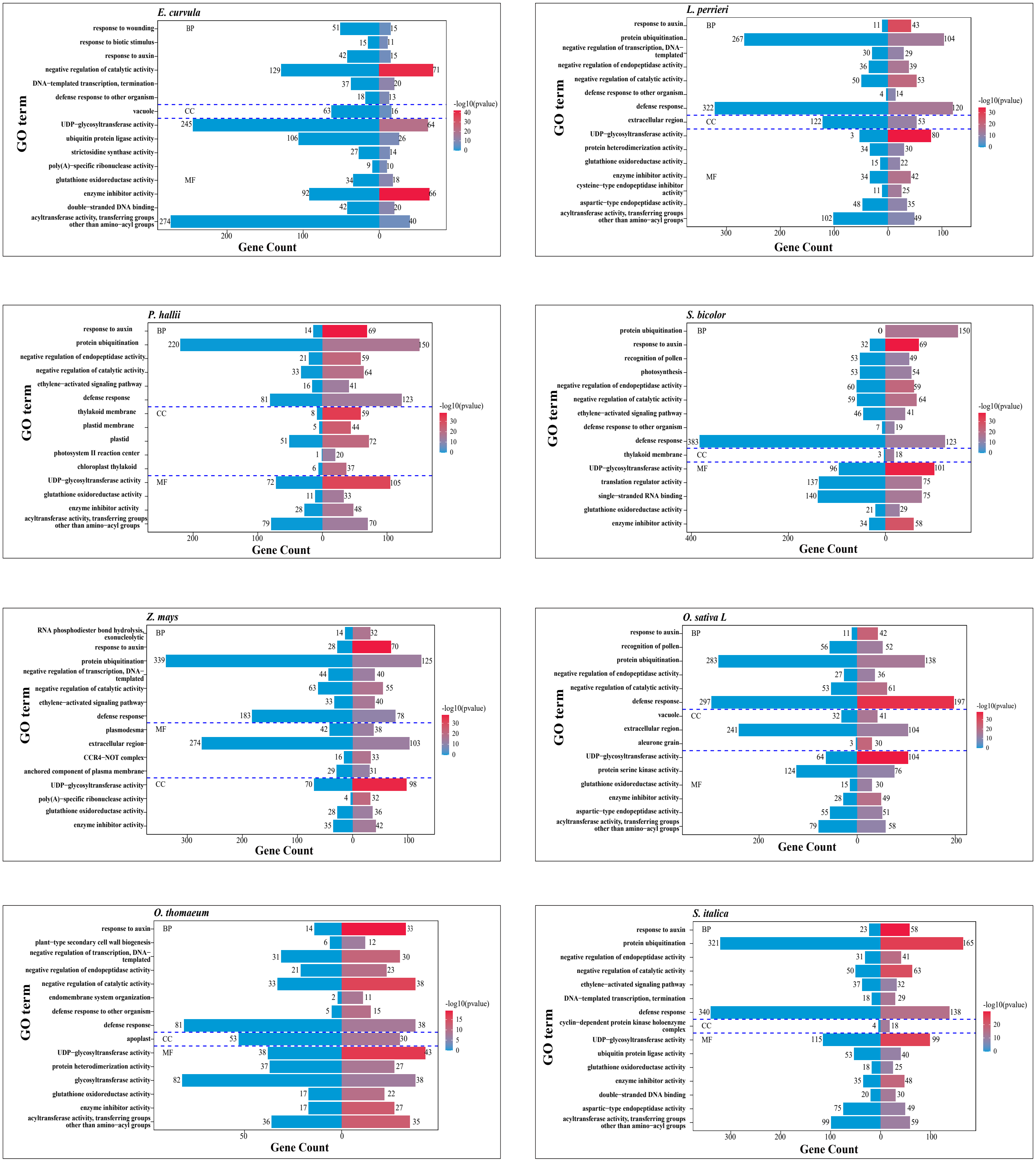

Supplementary Figure S7. Top 15 enriched Gene Ontology terms among the intronless genes (IGs, right) and multi-exon genes (MEGs, left).

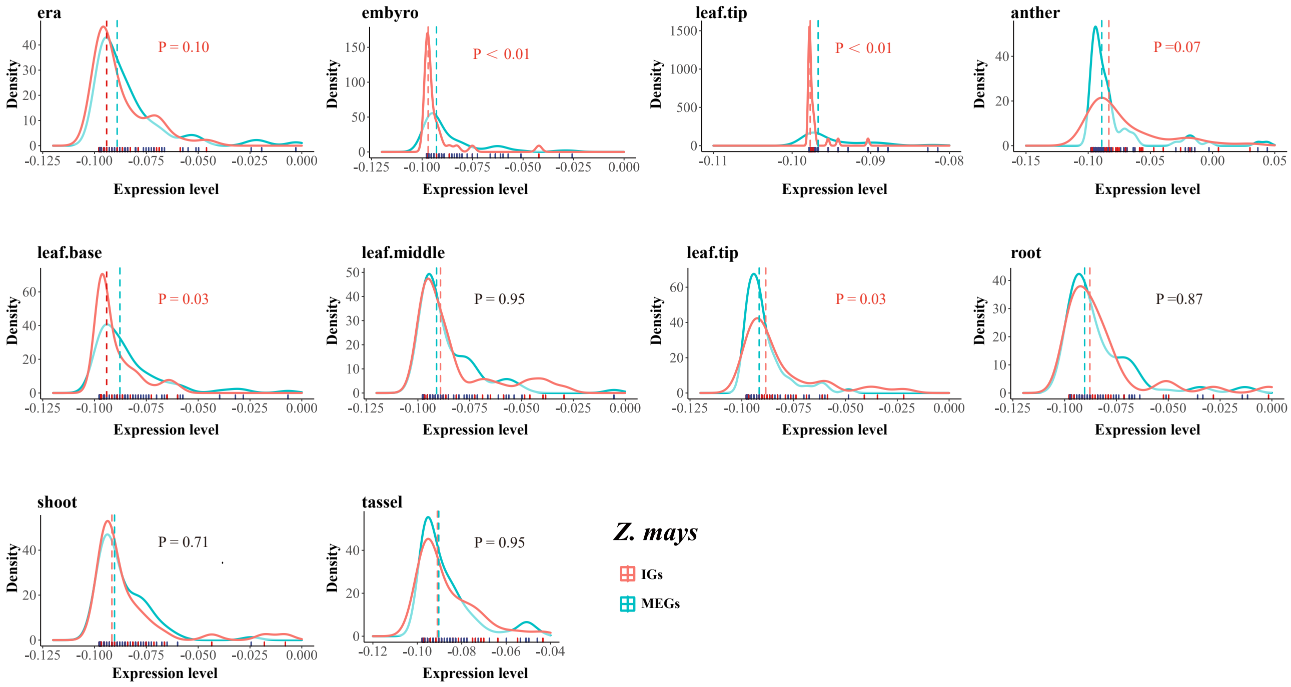

Supplementary Figure S8. Comparison of the intronless and multi-exon gene expression levels in maize tissues. The dotted line represents the median normalized expression level. The rug on the horizontal axis presents the density. The *P* value for the two-sided paired Wilcoxon sign test is provided. IGs, intronless genes; MEGs, multi-exon genes.
